# Synthesis of Pd/Ru Bimetallic Nanoparticles by *Escherichia coli* and Potential as a Catalyst for Upgrading 5-Hydroxymethyl Furfural Into Liquid Fuel Precursors

**DOI:** 10.3389/fmicb.2019.01276

**Published:** 2019-06-20

**Authors:** Jaime Gomez-Bolivar, Iryna P. Mikheenko, Rafael L. Orozco, Surbhi Sharma, Dipanjan Banerjee, Marc Walker, Rachel A. Hand, Mohamed L. Merroun, Lynne E. Macaskie

**Affiliations:** ^1^Department of Microbiology, Faculty of Sciences, University of Granada, Granada, Spain; ^2^School of Biosciences, University of Birmingham, Birmingham, United Kingdom; ^3^Dutch-Belgian Beamline, European Synchrotron Radiation Facility, Grenoble, France; ^4^Department of Chemistry, Katholieke Universiteit Leuven, Leuven, Belgium; ^5^Department of Physics, University of Warwick, Coventry, United Kingdom; ^6^Department of Chemistry, University of Warwick, Coventry, United Kingdom

**Keywords:** ruthenium bionanoparticles, Pd/Ru core-shells, 5-hydroxymethyl furfural conversion, 2,5-dimethyl furan synthesis, cellulose conversion

## Abstract

*Escherichia coli* cells support the nucleation and growth of ruthenium and ruthenium-palladium nanoparticles (Bio-Ru and Bio-Pd/Ru NPs). We report a method for the synthesis of these monometallic and bimetallic NPs and their application in the catalytic upgrading of 5-hydroxymethyl furfural (5-HMF) to 2,5 dimethylfuran (DMF). Examination using high resolution transmission electron microscopy with energy dispersive X-ray microanalysis (EDX) and high angle annular dark field (HAADF) showed Ru NPs located mainly at the cell surface using Ru(III) alone but small intracellular Ru-NPs (size ∼1–2 nm) were visible only in cells that had been pre-“seeded” with Pd(0) (5 wt%) and loaded with equimolar Ru. Pd(0) NPs were distributed between the cytoplasm and cell surface. Cells bearing 5% Pd/5% Ru showed some co-localization of Pd and Ru but chance associations were not ruled out. Cells loaded to 5 wt% Pd/20 wt% Ru showed evidence of core-shell structures (Ru core, Pd shell). Examination of this cell surface material using X-ray photoelectron spectroscopy (XPS) showed Pd(0) and Pd(II) and Ru(IV) and Ru(III), with confirmation by analysis of bulk material using X-ray absorption near edge structure (XANES) and extended X-ray absorption fine structure (EXAFS) analyses. Both Bio-Ru NPs and Bio-Pd/Ru NPs were active in the conversion of 5-HMF into 2,5-DMF but commercial Ru on carbon catalyst outperformed 5 wt% bio-Ru by fourfold. While 5 wt% Pd/20 wt% Ru achieved 20% yield of DMF the performance of the 5 wt% Pd/5 wt% Ru bio-catalyst was higher and comparable to the commercial 5 wt% Ru/C catalyst in a test reaction using commercial 5-HMF (>50% selectivity). 5-HMF was prepared by thermochemical hydrolysis of starch and cellulose with solvent extraction of 5-HMF into methyltetrahydrofuran (MTHF). Here, with MTHF as the reaction solvent the commercial Ru/C catalyst had little activity (100% conversion, negligible selectivity to DMF) whereas the 5 wt% Pd/5 wt% Ru bio-bimetallic gave 100% conversion and 14% selectivity to DMF from material extracted from hydrolyzates. The results indicate a potential green method for realizing increased energy potential from biomass wastes as well as showing a bio-based pathway to manufacturing a scarcely described bimetallic material.

## Introduction

Many types of living cells have the ability to template and form metallic nanoparticles (NPs) by reduction of soluble metal species. This has formed the subject of numerous studies and reviews (e.g., De Corte et al., 2012; Castro et al., 2014; Kulkarni and Maddapur, 2014; Singh, 2015; Singh et al., 2016). The goal is to develop alternative, facile, routes to the synthesis of industrially relevant catalysts using biomaterial scaffolds for catalytically active nanoparticles while preventing NP agglomeration and consequent loss of activity. Recent focus has moved toward the biosynthesis of bimetallic nanoparticles since these can have unique properties due to synergy of the metallic components. For example in Pd/Au the formation of Pd^δ+^/Au^δ–^ was suggested to underlie the superior catalytic activity of the bimetallic (Gao and Goodman, 2012). However, synthesis of bimetallic NPs by chemical routes is more difficult than for monometallic counterparts. Various preparation methods of bimetallic nanostructures have been reviewed (e.g., Zaleska-Medynska et al., 2016) and a variety of shapes, properties and catalytic activities has been obtained. However, biosynthetic routes are relatively unexplored, despite the potential for applying the tools of synthetic biology to obtain targeted NP manipulation (Torgeman, 2017).

An early example of bio-Pd/Au NPs was reported by Deplanche (2008) and this bio-catalyst, made on *Escherichia coli* and *Cupriavidus necator*, was applied in two respective catalyses: partial oxidation of benzyl alcohol to benzaldehyde (Deplanche et al., 2011) and reduction of *p*-nitrophenol (Hosseinkhani et al., 2012). The former, together with bio-Pd/Au made by *Desulfovibrio desulfuricans* (Tran et al., 2012) had a core-shell structure (Au core/Pd shell). This structure is formed by initially depositing “seeds” of Pd(0) nanoparticles with enzymatic assistance involving hydrogenases (Mikheenko et al., 2008; Deplanche et al., 2010). The resulting Pd(0) reduces Au(III) galvanically and oxidized Pd species migrate outward from the core of neo-Au(0) followed by their chemical reduction under hydrogen to form a shell of Pd(0) (Deplanche et al., 2012). Bio-Pd/Au core shells form inside the bacterial cytoplasm (Supplementary Figure S1), which implies uptake and processing mechanisms for these heavy metals, that have no known biological function. Although the bacteria remain metabolically competent during Pd(0) “seeding,” as shown by the use of flow cytometry (Omajali et al., 2018), the routes by which the Pd(0) “seeds” are localized and then develop from initial Pd-nuclei is still unknown, despite that these are key to the patterning of the subsequent bimetallic. Following formation of the Pd “seeds” cell viability is lost rapidly, although hydrogenase activity persists for several hours (Mikheenko et al., 2008). The use of dead cells [and retention of the NPs upon them (Bennett et al., 2013)] ensures acceptability of the nanomaterial while mitigating against NP release into the environment. The need to supply Pd(II) in acidic solution (10 mM HNO_3_), was shown by previous optimization studies; the function of the acid is to protonate the polyanionic cell surface to permit access of the PdCl_3_^–^ ion that predominates in solution. Importantly, deposition of the second metal is an abiotic process, which enables metal recovery from highly acidic solutions following “seeding” with Pd(0) under physiologically compatible conditions (Murray et al., 2018).

Using a similar approach, the formation of bimetallic bio-Pd/Pt NPs was recently reported (Murray et al., 2018). These were active in the catalytic reduction of Cr(VI) (Murray et al., 2018) and in the selective hydrogenation of soybean oil (Murray et al., 2018) and 2-pentyne (Murray et al., 2017) as well as in the catalytic upgrading of heavy oils from Canadian oilsands (Omajali et al., 2017), and oils produced by thermochemical processing of wet biomass (Kunwar et al., 2017). However, the arrangement of the metallic components in the NPs (e.g., alloys or core-shell structures) was not reported.

With a developing global focus on sustainable energy and green chemistry Pd/Ru bimetallics have been highlighted in these areas but study of Pd/Ru is neglected in comparison with Pd/Au. Raja et al. (1999) showed that the hydrogenation of hex-1-ene to *n*-hexane was several orders of magnitude higher via use of Pd_6_Ru_6_ clusters than with Pd alone. Later, Qui et al. (2006) showed higher conversion and selectivity in hydrogenation of cinnamyl alcohol using Pd/Ru catalyst compared to that obtained by using single metals. Luo W. et al. (2015) reported catalytic hydrogenation of levulinic acid by a Pd/Ru bimetallic alloy; here, the metals were randomly dispersed and the high catalytic activity was attributed to dilution and isolation of Ru by Pd (Kyriakou et al., 2012). Boucher et al. (2013) had previously attributed highly selective hydrogenations to isolated Pd atoms. On the other hand, oxidation of formic acid (Liu et al., 2012) was reported, and also oxidation of ethanol, the latter using Pd-Ru bimetallic-NPs on carbon; this catalyst comprised a mix of Pd metal, Ru oxides and Pd oxides (Monyoncho et al., 2015). A Pd-overlayer enhanced the activity of Ru-nanotubes in hydrogen oxidation (St. John et al., 2015). Clearly, the activity for a certain reaction relates to the metal arrangement in the NPs but production of Pd/Ru core-shell structures is neglected. Modulating fcc and hcp ruthenium on the surface of a Pd-Cu alloy produced a core-shell (Yao et al., 2016) but the catalytic activities of hcp-dominated Ru-Cu NPs and fcc-dominated Ru showed opposing results in hydrogenations of 4-nitrochlorobenzene and styrene according to the predominant type of Ru. This highlights the potential to moderate selectivity according to the bimetallic fine structure but also cautions that the outcome of a reaction may be difficult to achieve if the metal arrangement is not controlled. Biomanufacture of Pd/Ru NPs is not yet reported in the literature. An initial study (Omajali, 2015) suggested this route for making bimetallic NPs for the catalytic conversion of 5-hydroxymethyl furfural (5-HMF) to 2,5 dimethyl furan (DMF) but no NP characterization was performed.

5-HMF is a derivative of glucose, fructose (van Putten et al., 2013) or cellulose under thermochemical degradation (Román-Leshkov et al., 2007). The product, DMF (Lei et al., 2014; Nagpure et al., 2015), is a “platform” precursor of plastics and also of “drop in” fuels (Lei et al., 2014; Nagpure et al., 2015). “Drop-in” biofuels are defined as “liquid bio-hydrocarbons that are functionally equivalent to petroleum fuels and are fully compatible with existing petroleum infrastructure” (Karatzos et al., 2014). Working toward higher yields and selectivity toward DMF, studies have focused on “classical” mono and bimetallic catalysts including Pd and Ru (Hansen et al., 2012; Nishimura et al., 2014; Zu et al., 2014; Luo J. et al., 2015). Study of bacterially derived Pd/Ru NPs is a new development. Omajali et al. (2019) showed the potential of cells of the Gram-positive bacterium *Bacillus benzeovorans* to make bio-Pd/Ru bimetallic structures using the same approaches as described above for bio-Pd/Au and bio-Pd/Pt. Most of the work on bio-NP catalysts has used Gram-negative bacteria. Deplanche et al. (2014) and Zhu (2014) noted that bio-Pd catalysts supported on typical Gram-positive cells were less active catalytically than those on Gram negative bacteria. Hence the primary aim of this work was to evaluate the potential for the use of the paradigm Gram negative *E. coli* to synthesize NPs of bio-Ru and bio-Pd/Ru and evaluate their potential for the catalytic upgrading of 5-HMF to DMF. In order to move toward real-life application the upconversion of 5-HMF derived from thermochemical hydrolysis of starch and cellulose was also evaluated.

The use of *E. coli* is attractive as this ubiquitous organism is readily grown at scale and waste *E. coli* cells grown for another primary process (biohydrogen production) were successfully used in “second life” to make bio-Pd catalyst for hydrogenation (Zhu et al., 2016) and in fuel cells (Orozco et al., 2010), while the ability to fabricate the metallic catalyst from liquid wastes (Yong et al., 2010, 2015; Murray et al., 2017) has positive implications for both economy and sustainability.

## Materials and Methods

### Bacteria, Growth Conditions, and Chemicals Used

*Escherichia coli* strain MC4100, grown as described by Deplanche et al. (2012), was harvested in mid-logarithmic phase (OD_600_ of 0.7–1.0) by centrifugation (9,000 × *g*, 15 min, 4°C), washed three times (20 mM MOPS-NaOH buffer, pH 7.0) and routinely stored as a concentrated suspension overnight (4°C). The cell dry weight was estimated from a previously determined OD/dry weight conversion.

Commercial metal salts (Na_2_PdCl_4_ and RuCl_3_) were from Sigma-Aldrich, as were 5 wt% Pd and 5 wt% Ru on carbon catalysts and commercial 5-HMF (≥99%) and 2,5-DMF (99%).

### Preparation of Monometallic and Bimetallic Bionanoparticles (Bio-NPs)

For monometallic bio-Ru cell suspension was diluted into 2 mM Ru (III): RuCl_3_.2H_2_O solution (pH 2, in 10 mM HNO_3_) to the required biomass/metal ratio for the desired loading (5 wt%) and left for 30 min (30°C) for metal uptake by the cells. H_2_ was bubbled through the suspension for 1 h and left for 96 h (sealed bottle; 180 rpm agitation; 30°C). Monometallic bio-Pd (5 wt%) was made similarly, according to Deplanche et al. (2012).

Synthesis of bimetallic Pd/Ru used, sequentially, a 2 mM Pd (II) and then a 1 mM Ru (III) solution (in 10 mM HNO_3_) by the method of Deplanche et al. (2012) with modifications: 2 mM Pd (II) solution was reduced to Pd(0) on the cells under H_2_ (30 min; complete removal (by assay) of residual soluble metal) to give 5 wt% bio-Pd(0). The bio-Pd(0) was washed twice (distilled water, DW), and added as a concentrated suspension into 1 mM Ru (III) solution (final concentration; volume was adjusted to give the required final metal loading on cells) to give a final loading of (nominally) 5 wt% Pd/5 wt% Ru or 5 wt% Pd/20 wt% Ru. The bio-Pd/Ru mixture was left to stand then saturated with H_2_ (as above; 180 rpm, 30°C; 96 h). The presumptive bio-NPs were washed three times (DW) and once with acetone (9,000 × *g*, 15 min, 4°C), air-dried and ground manually. The actual Ru loadings were determined by difference via assay of residual soluble Ru(III) by the stannous chloride method (Charlot, 1978); Pd was completely removed in the first step and was retained on the cells (as determined by assay of wash solutions).

### High Resolution Scanning-Transmission Electron Microscopy (STEM) With HAADF (High-Angle Annular Dark Field) Detector, Energy Dispersive X-Ray Analysis (EDX), and Determination of Lattice Spacing

Where cell sections were to be examined, fresh preparations were fixed [2.5% (w/v) glutaraldehyde fixative in 0.1 M cacodylate buffer, pH 7.2; 2 h at 4°C], washed three times with the same buffer and stained (1% aq. osmium tetraoxide). For TEM thin samples were prepared as described previously (Deplanche et al., 2012). Electron opaque deposits were examined by EDX with peaks sought corresponding to X-ray emission energies of Ru and Pd. STEM and EDX were done using a FEI image Cs-corrector configuration Titan^TM^ G2 60-300 STEM microscope equipped with HAADF detector, accelerating voltage of 300 kV. Lattice spacings were determined using “ImageJ” (Abramoff et al., 2004) through profiling of high resolution HAADF-STEM images.

### X-Ray Photoelectron Spectroscopy (XPS) of Cell Surfaces

Subsamples (a few mg) were retained and air-dried. Surface chemical composition and oxidation state analyses were done by XPS via published methods (Omajali et al., 2017) using a Kratos Axis Ultra DLD spectrometer (Kratos Analytical). The samples were illuminated using an Al Kα x-ray source and the photoelectrons were collected using a hemispherical electron analyzer. Survey spectra were recorded using a pass energy of 160 eV, with the pass energy reduced to 20 eV for acquisition of the core level spectra (resolution approx. 0.4 eV). The samples were insulating, therefore a charge neutralizer was used to prevent surface charging with a low energy electron beam directed onto the sample during XPS data acquisition. Measurements were made at room temperature and at a take-off angle of 90°, to probe a depth of approx. 5–10 nm to examine bio-NPs bound to the outermost cell surfaces. Generated data were converted into VAMAS format and analyzed using the CasaXPS package (Fairley, 2013) employing Shirley backgrounds, mixed Gaussian-Lorentzian (Voigt) lineshapes and asymmetry parameters where appropriate. All binding energies were calibrated to the C 1s peak originating from C-H or C-C groups at 284.8 eV.

### X-Ray Absorption Spectroscopy (XAS) Analysis

This synchrotron radiation based technique was used to determine the local coordination of Pd and Ru in the biogenic Pd/Ru NPs samples. Pd and Ru K-edge XAS spectra were acquired at the Dutch-Belgian Beamline (DUBBLE) beamline at the European Synchrotron Radiation Facility (ESRF), Grenoble (France), using a Si(111) monochromator operating in fixed-exit mode. Data were acquired using Ar/He filled ionization chambers (transmission mode). The energies were calibrated by measuring the Pd and Ru K-edge transmission spectra of Pd and Ru foils and were calibrated to 24350 and 22117 eV, respectively. Samples (Ru and Pd/Ru-loaded cells) were examined as dry samples (powder, a few mg). Data were processed using the ATHENA code (Ravel and Newville, 2005) with subtraction of background via a pre-edge linear function. Atomic absorption was simulated with a square-spline function. The amplitude reduction factor was held constant at 1.0 for the FEFF8 calculation and extended X-ray absorption fine structure (EXAFS) fits, with the shift in threshold energy, ΔE0, varied as a global parameter. The theoretical scattering phase and amplitude functions used in data analysis were calculated using FEFF8 (Ankudinov et al., 1998). For the Pd edge EXAFS spectra, data for phase-shifts and backscattering amplitudes were obtained from reference materials of PdO (Pd–O scattering) and Pd foil (Pd–Pd scatterings). For the Ru edge EXAFS spectra, data for phase-shifts and backscattering amplitudes were obtained from RuO_2_ (Ru–O scattering), Ru foil (Ru–Ru scatterings), and RuCl_3_ (Ru–Cl scattering) reference compounds.

### Preparation of 5-HMF via Thermochemical Hydrolysis of Starch and Cellulose

Methods for thermal hydrolysis were as reported previously (Orozco et al., 2012). For starch/cellulose hydrolysis the batch reactor system comprised a bench top reactor (100 ml; Parr series 4590; pressure 200 bar; temperature 350°C) of Type 316 stainless steel equipped with a heat/agitation controller (Parr 4848). Temperature and pressure were measured from inside the reactor and maintained within 1 bar and 0.1 K, respectively.

For hydrolysis the material (starch (7.2 g, from potato powder; Sigma-Aldrich) or cellulose (5.1 g, Sigma-Aldrich) was suspended in de-ionized water (final reactant volume of 60 ml for starch; 120 g/l and 70 ml for cellulose; 72.9 g/l) or as otherwise stated) and charged into the reactor for hydrolysis (head space ∼120 ml). The reactor was sealed, purged with N_2_ three times, pressurized (30 bar), heated to the set-point temperature (220°C for starch, 260°C for cellulose; agitation 300 rpm), held for 15 min and cooled by submersion in cold water. The hydrolyzate was separated (after depressurization) from solid residue (vacuum filtration; filter paper Fisherbrand QL100) or by centrifugation (10,000 rpm; 10 min). Hydrolyzates and samples were kept at 4°C prior to analysis. The reactions were repeated as required to produce sufficient pooled starch and cellulose-derived 5-HMF for the upconversion tests.

Hydrolyzates were analyzed using a GC (Shimadzu, 2010 equipped with an autosampler AOC-20S, a FID detector and ZB-Wax column (30m × 0.25 mm × 0.25 μm); injection volume 1 μl; inlet temperature 260°C; injector temperature 300°C; detector temperature; 300°C, inlet pressure 100 KPa; split ratio of 100:1 with H_2_ carrier gas at a flow rate of 1 ml/min). The heating gradient was 0 min GC temp 100°C; 10 min GC temp 200°C; 22 min GC temp 200°C; and 25 min GC temp 250°C. Reaction residues were not quantified nor analyzed.

### Solvent Extraction of 5-HMF Using 2-Methyltetrahydrofuran (2-MTHF)

The method for 5-HMF extraction was based from the experimental determination of partition coefficients under batch and continuous conditions according to Blumenthal et al. (2016). The mass transfer of 5-HMF from the aqueous to the organic phase is faster at 60°C and concentrations of 5-HMF in the range between 1–5 wt% in the aqueous feed had little effect on the partition coefficients. Therefore, the produced starch and cellulose hydrolyzates, respectively, were mixed in equal volumetric proportions with 2-MTHF (organic extraction solvent) in an Erlenmeyer flask at 200 rpm and 60°C using a magnetic stirrer and a temperature-controlled water bath (25 min). After extraction aqueous and organic phases were separated using a separation funnel: the top organic phase was “supernatant” and the bottom aqueous phase was “hydrolyzate.” Both phases were sampled and kept at -20°C before analysis by GC.

Solvent extraction efficiency was calculated using the following formula:

Extractionefficiency(%)=moles⁢of⁢ 5⁢-⁢HMF⁢in⁢supernatantmoles⁢of⁢ 5⁢-⁢HMF⁢in⁢hydrolyzate×100

### Catalytic Conversion of 5-Hydroxymethyl Furfural to 2,5-Dimethyl Furan

Starch and cellulose derived 5-HMF were obtained via hot compressed water treatment (Orozco, 2012; Orozco et al., 2012) followed by solvent extraction of 5-HMF using MTHF (as above). The catalytic transfer hydrogenation reactions used a 100 ml Parr series 4590 bench top reactor of Type 316 stainless steel equipped with a heat/agitation controller (Parr 4848). Three sets of experiments were carried out: set 1 (commercial 5-HMF); set 2 (starch-derived 5-HMF); and set 3 (cellulose-derived 5-HMF). For set 1 the reactor was charged with 250 mg of 5-HMF in 25 ml of MTHF (80 mM 5-HMF solution); for sets 2 and 3 volumes of 28 and 50 ml of 5-HMF in MTHF were extracted from starch and cellulose hydrolyzates, respectively. In all sets a weight ratio of 2.5:1 of 5-HMF:catalyst was added to the reactor. The reactor was sealed, purged three times with H_2_ (50 bar), pressurized with H_2_ (50 bar), and heated (260°C; 2 h; 500 rpm). After the reaction (time as determined by prior tests), the reactor was quenched to 35–40°C in a water bath and the reaction mixture was filtered (Fisherbrand QL100 filter paper). Samples were stored at -20°C before analysis using a GC-FID for quantification and a GCMS-QP2010s for compound identification. All GC-FID analysis was performed on a Shimadzu GC2014 GC equipped with a Shimadzu AOC-20i autosampler. The carrier gas was hydrogen, supplied by an external hydrogen generator (Parker). The GC was fitted with a Restek Stabilwax-DA column (30 m length, 0.32 mm ID, and 0.25 μm film thickness). The injection volume was 1 μl with a 39 split ratio. The inlet temperature was 250°C. The detector was a flame ionization detector (FID) with a flame temperature of 300°C, and a sampling rate of 40 ms. The heating profile was 60°C for 2 min then heated to 200°C at 5°C/min min followed by further heating to 240°C at 15°C/min where it remained for a further 3 min. Analysis was carried out using Shimadzu GCsolutions software. Calibration curves were third order between 80 and 0.4 mM.

All GC-MS analysis was performed on a Shimadzu GCMS-QP2010s equipped with a Shimadzu AOC-20i autosampler. The carrier gas was helium. The GC was fitted with a Restek Rxi-1ms column (15 m length, 0.25 mm ID and 0.25 μm film thickness). The injection volume was 1 μl with a −1 split ratio. The inlet temperature was 250°C. The detector was a single quadrupole mass spectrometer in electron ionization mode. The detector and interface temperatures were 250°C. The detector acquisition mode was scanning between 40–400 m/z, with a scan every 300 ms. The solvent cut time was 1 min. The heating profile was 60°C for 2 min then heated to 200°C at 5°C/min followed by further heating to 240°C at 15°C/min where it remained for a further 3 min. Analysis was carried out using Shimadzu GCMS Real-Time Analysis and Shimadzu GCMS Post Run Analysis software.

Conversion of 5-HMF and yields of DMF were calculated as follows:

5-HMFconversionin(%)=  (1-moles⁢of⁢ 5⁢-⁢HMF⁢in⁢productsstarting⁢moles⁢of⁢ 5⁢-⁢HMF)× 100

2,5-DMFyield(%)=  (moles⁢of⁢ 2,5⁢-⁢DMF⁢in⁢productsstarting⁢moles⁢of⁢ 5⁢-⁢HMF)× 100

2,5-DMFselectivity(%)=  (molesof 2,5-DMFinproducts)starting⁢moles⁢of⁢ 5⁢-⁢HMF-final⁢moles⁢of⁢ 5⁢-⁢HMF)× 100

Other products were not identified or quantified.

## Results and Discussion

### Uptake of Pd(II) and Ru(III) by the Cells and Formation of Bio-Pd and Bio-Ru NPs

Initial studies using Pd(II) showed its rapid, complete removal from solution by *E. coli* (Deplanche et al., 2010) and conversion into Pd(0)-NPs, both at the cell surface and intracellularly, within 30 min (Supplementary Figure S2). In contrast, with Ru(III), only ∼50% of the Ru(III) was removed, even after 96 h (Table 1); hence, the nominally 5 wt% Ru was actually 2.6% of the cell dry weight. Thermogravimetric analysis of *D. desulfuricans* biomass (Omajali et al., 2017) showed that typically more than 50% of the material remains at above 600°C, comprising residual carbon and mineral components. Hence, for the purpose of this comparison, the 2.6 wt% bio-Ru (on air dried cells) and commercial 5 wt% Ru/C catalysts are assumed to be broadly comparable in terms of metal/carbon but the dosing of catalyst metal into the catalytic tests (see later) would be ∼half in terms of metallic component of the biomaterial on a comparable weight basis. However, the two catalysts are probably not comparable in terms of available catalyst surface; attempts to establish the surface area of bio-Pd by standard sorption methods were unsuccessful (Bennett and Macaskie, unpublished).

**TABLE 1:** Materials examined in this study prepared on cells of *Escherichia coli*.

		**Nominal wt%**		**Actual wt%**		**Metal loading per catalyst (actual, wt%)**
			
		**Pd**	**Ru**	**Pd**	**Ru**	
I	5% bio-Ru	0	5.0%	0	^*^2.6%	2.6%
II	5%/5% bio-Pd/Ru	5.0%	5.0%	5.0%	^*^4.7%	9.7%
III	5%/20% bio-Pd/Ru	5.0%	20%	5.0%	^*^17.5%	22.5%

^*^The actual metal loading was determined by difference from the Ru(III) provided and that found in the spent solution by assay of the spent solution by assay (see text). Ru(III) sample (0.2 ml, aq.) was added to 0.8 ml of stannous chloride (29.9 g SnCl_2_ in 500 ml conc. HCl) and incubated at 30°C (30 min). Ru(III) was estimated at A_400_ with reference to a Ru(III)-calibration similarly determined and was linear in the region of interest. Note that atomic weights of Pd and Ru are 106.4 and 101.1, respectively; hence sample II was near-equimolar.

Examination of 5 wt% bio-Pd (the “seeds” to promote Ru deposition) showed that Pd-free cells had no nanoparticles. The deposition of Pd(0) on/in the challenged cells was very similar to that reported for bio-Pd made on *D. desulfuricans* (Omajali et al., 2015 and Supplementary Figure S2). A full characterization of the bio-Pd on *E. coli* will be was described by Gomez-Bolivar et al. (2019).

**FIGURE 1:**
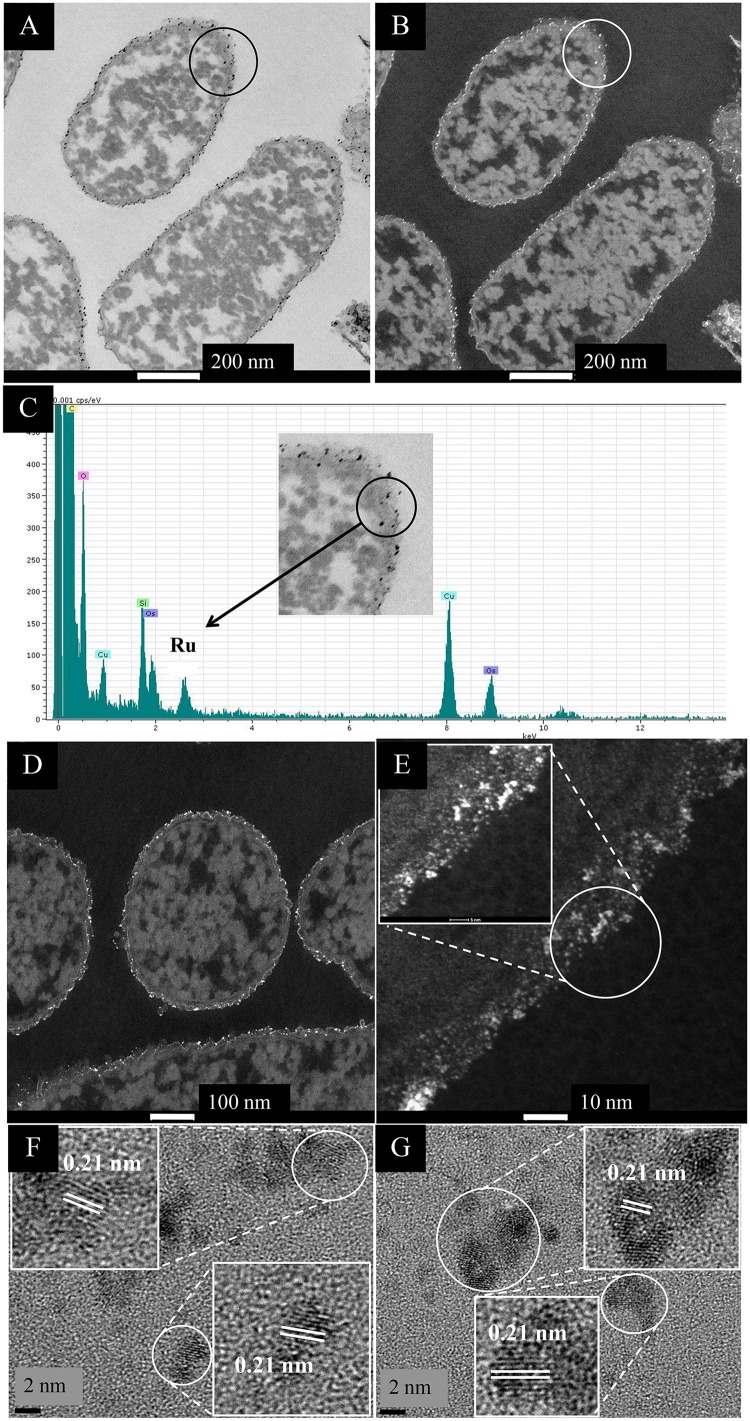
EM study of *E. coli* MC4100 cells loaded to 2.6 wt% of Ru. **(A,B)** STEM/HAADF images of cell sections. For comparison cells with no added metal are shown in Supplementary Figure S2. Magnification of the circled section (inset) shows the presence of nanoparticles located in the membrane (**C**, inset.) EDX analysis confirmed the presence of Ru in the cell surface NPs **(C)**. Cu is from the EM grid and Os from the stain. Bars are 100 nm **(A,B)**. HAADF image is shown enlarged **(D,E)** revealing heterogeneity of Ru-NP sizes **(E)** and NP localization only in the periplasm (width of periplasm < 35 nm). HR-TEM analysis of the circled area in panel **(E)** revealed consistent lattice spacing of 0.21 nm **(F,G)** which can be attributed to either Ru metal or RuO_2_ (see text).

In contrast, cells challenged with Ru(III) alone showed localization of electron opaque NPs detectable only at the cell surface (Figures 1A–E) and also in material extruded from the cell surface both at 2.6 wt% Ru (not shown) and in cells loaded with Ru to (nominally) 20 wt% Ru (the actual loading here was not determined) (Supplementary Figure S3). Analysis of the cell surface (Figure 1C) and exuded material (Supplementary Figure S3) using EDX confirmed the presence of Ru, while HR TEM (Figures 1F,G) showed discrete NPs of size ∼2–3 nm, with lattice spacings of 0.210 nm, which may be assigned to the {101} face of Ru metal (0.205 nm: Ghosh and Chen, 2008). However, other studies (Kim et al., 2001) concluded that RuO_2_ forms as a surface layer by epitaxial growth on the surface of Ru in a lattice-matched manner; indeed, Leng et al. (2014) attributed a lattice fringe of 0.205 Å to the {210} face of RuO_2_ synthesized on graphene. *In situ* synchrotron X-ray diffraction showed the direct transition of amorphous Ru(OH)_3_.H_2_O to crystalline RuO_2_ NPs, i.e., the evolution of Ru(IV) from Ru(III) (Park et al., 2015). The hydrolysis behavior of the Ru^3+^ ion in the current study would be suppressed at the acidic pH used for metal uptake (as described by Deplanche et al., 2012) but following metal exposure (under H_2_) the cells were washed in water and left in air prior to analysis and hence oxidation of any residual Ru(III) in air cannot be precluded. As some of the X-ray emission energies of Ru and Cl overlap [respectively, keV 2.56 (Lα) 2.68 (Lβ) and 2.62 (KαY); 2.81 (Kβ) keV] elemental mapping cannot preclude deposition of RuCl_3_ [in contrast the emission lines of Pd are (keV) 2.84 (Lα1), 2.99 (Lβ1), 3.17 (Lβ2), and 3.33 (Lγ)]. Hence, XPS analysis of metal and chloride speciation at the cell surface was performed (see later).

Ru was not apparently taken up into the cytoplasm (Figure 1 and Supplementary Figure S3) and the extruded material is suggested to be of cell surface origin, since outer membrane vesicles containing Ru were visible (see later). The Ru-deposition extended through the thickness of the cell wall layer (Figure 1E). There is little information on the interactions of Ru(III) with living cells but Ru-complexes are very common, e.g., ruthenium-amine complexes are reported to have antitumor activity (e.g., Lima et al., 2014) while a recent report (Luo et al., 2018) describes the formation of Ru(III) complexes with collagen, a structural protein. In bacteria it seems likely that incoming Ru(III) is intercepted by amine groups of the periplasmic peptidoglycan, while outer membrane proteins would also form binding sites for Ru(III); we surmise that the incoming Ru(III) is intercepted and held by ligands in the cell surface layers.

### Deposition of Ru and Pd/Ru by Cells of *E. coli*

In contrast to the above, when loading the cells with 5 wt% Pd/5 wt% Ru or 5 wt% Pd/20 wt% Ru the Ru(III) was removed from the solution by 94 and 88%, respectively (Table 1). Clearly “seeding” with 5 wt% Pd(0) promotes deposition of Ru as compared to challenge with Ru(III) alone. The nominal and actual loadings of Ru on the cells are shown in Table 1; for convenience the bimetallic samples will be described as “low-Ru” (5 wt% Pd/5 wt% Ru) and “high Ru” (5 wt% Pd/20 wt% Ru), respectively, a key difference being the greater proportion of Pd atoms in the samples with less Ru.

### Examination of Low-Ru Bimetallic by HRTEM and HAADF and Elemental Mapping

**FIGURE 2:**
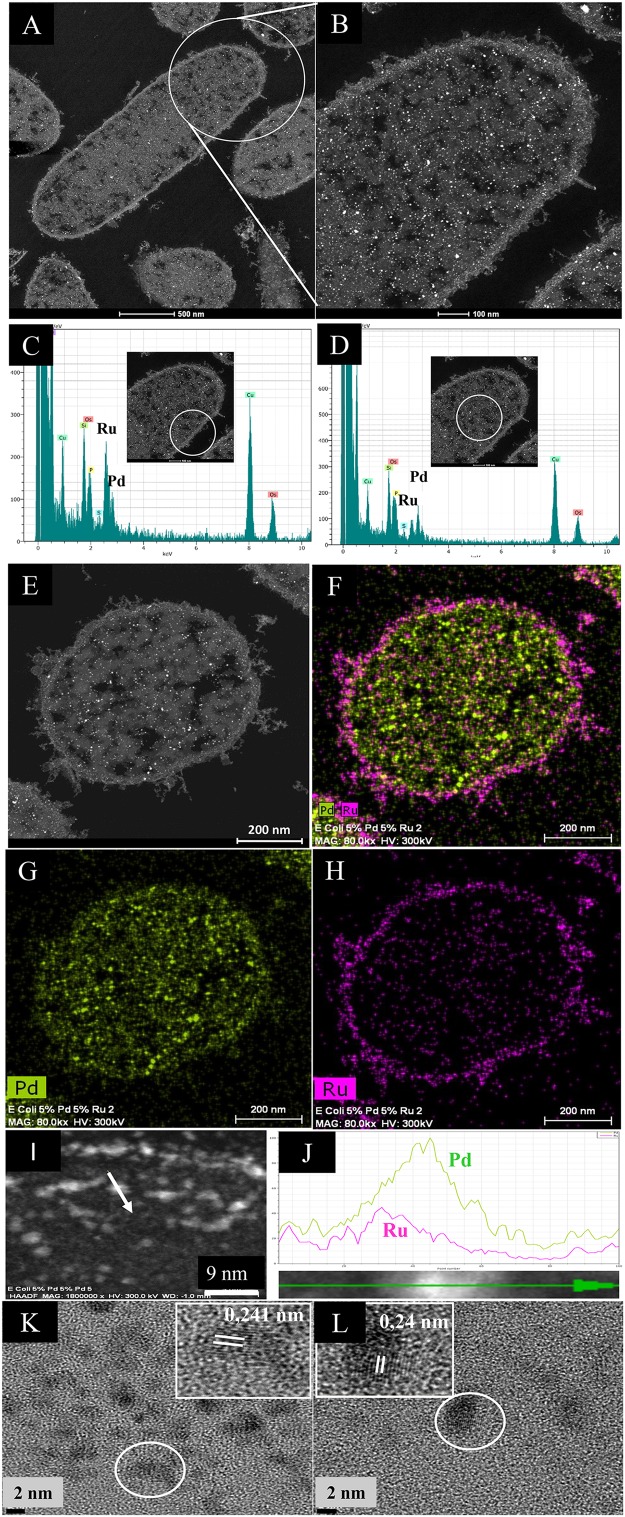
HAADF/STEM micrographs of cell sections **(A)** and magnified view. Bars are 500 nm **(B)** of 5 wt% Pd/5 wt% Ru NPs. Bars are 100 nm. EDX is shown of the cell surface **(C)** and intracellular **(D)** regions. A single cell **(E)** is shown mapped for areas of Pd **(G)** and Ru **(H)** localization and co-mapped to show distribution of the two elements **(F)**. Bars are 200 nm. An enlarged image of panel **(F)** is shown in Supplementary Figure S4 to show overall lack of co-mapping of the two elements on visual inspection but also that intracellular Ru is very evident. An example NP in the cell surface region (**I**, arrowed; scale bar is 9 nm) was analyzed by transect **(J)** to show association between Pd (green) and Ru (magenta), especially evident on one side of the NP as a skewed distribution. The green arrow (bottom) shows distance across the transect (as a percentage 0–100%) and the *Y*-axis is counts (arbitrary). **(K,L)** HRTEM images of single NPs from membrane-bound **(K)** and cytoplasmic **(L)** NPs showing lattice fringes. Scale bar is 7 nm.

Challenge of the cells with Pd(II) and Ru(III) individually suggested that, while the former entered the cells and formed intracellular deposits, Ru deposition was confined to the surface layers (above) and hence it was implied that only surface-located Pd(0) would be able to “seed” the formation of structured bimetallics. However, intracellular NPs were visible (Figures 2A,B); these, and also the surface-located NPs, contained both Pd and Ru (Figures 2C,D), with an enrichment of Ru in the latter region (Figure 2C). Elemental mapping (Figures 2E–H) confirmed the uniform distribution of Pd (Figure 2G), surface-enrichment of Ru, the presence of small intracellular Ru-NPs and putative membrane vesicles containing Ru-NPs (Figure 2H and Supplementary Figure S4). Intracellularly, Pd-NPs predominated (Figure 2F). While some areas of the cell contained Pd-NPs only, the Ru-NPs were located mainly alongside Pd-NPs (Supplementary Figure S4) although an association between them was not proved.

To prove an association between Pd and Ru a cell surface NP transect (arrowed in Figure 2I) was analyzed (Figure 2J), showing an asymmetric hybrid structure with Pd/Ru at one side and Pd-enriched at the other, corresponding to a sparse region of Ru. The predominance of Pd in the NPs was confirmed by the lattice fringes (0.24 nm: Figures 2K,L) assigned to Pd{111} facets (Omajali et al., 2015) in both the cell surface and intracellular NPs. The size of the NPs was ∼3 nm but a NP size distribution analysis was not attempted due to the difficulty of setting the NP boundary due to the indistinct nature of the NPs. Some size heterogeneity is apparent (Supplementary Figure S4) but it is not certain if the larger NPs are simply agglomerations of smaller ones. The mean NP size (for bio-Pd) was reported as 1.4 nm in *D. desulfuricans* (Omajali et al., 2015), while that for *E. coli* was similar, at 1.3 nm (Gomez-Bolivar et al., 2019) and will be detailed in a subsequent publication.

### Examination of High-Ru Bimetallic by HRTEM and HAADF and Elemental Mapping

**FIGURE 3:**
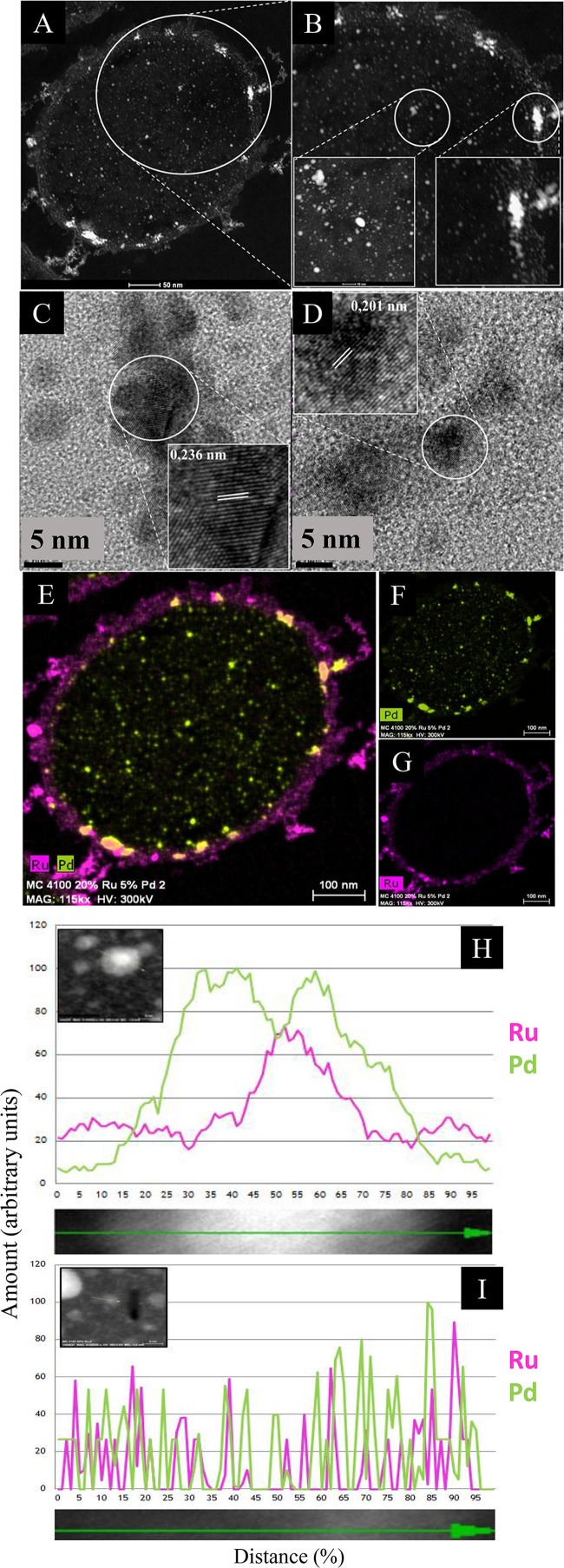
HAADF/STEM micrographs of cell sections showing Pd/Ru NPs (5 wt% Pd/20 wt% Ru). **(A)** A higher magnification panel **(B)** shows NPs located in the cell surface layers (**B**, inset right) and in the bulk region (**B**, inset left). Lattice spacing of example NPs in cell wall layers and intracellularly are shown in, respectively **(C,D)**. Elemental maps (by EDX) of cells show co-localization of Pd and Ru **(E)** and individually Pd **(F)** and Ru **(G)**. Elemental maps (by EDX) of a single nanoparticle showing a core/shell structure **(H)** and random distribution **(I)** of Pd and Ru.

Figure 3 shows the NPs produced at the higher loading of Ru. In contrast to the low-Ru samples (above) no intracellular Ru NPs were apparent by electron microscopy (Figure 3G and Supplementary Figure S5) although some Ru was detected intracellularly by EDX (Supplementary Figure S6). Small and larger intracellular NPs were visible; the latter contained more Pd (Supplementary Figure S6). The reason for the apparently low cellular uptake of Ru and lack of Ru-NPs was not investigated but the higher dose of Ru was possibly lethal to the cells. The observation of intracellular Ru (Supplementary Figure S6) but not NPs (Figure 3E) also raises the question as to the actual role of Pd(0) seeds in the reduction of Ru(III) (assumed on the basis of earlier work using bio-Pd/Au: Deplanche et al., 2012) as a similar result to the low-Ru preparation (above) would be expected.

For catalysis the surface bound material would be more relevant and this was examined further. Lattice fringes (0.236 nm; Figure 3C) would correspond to Pd(0) {111}. Other images (not shown) confirmed lattice fringes of 0.240 nm, attributed to Pd {111} facets (Omajali et al., 2015) or possibly to the {110} plane of RuO_2_ (0.231 nm: Soin et al., 2012), while the 0.201 nm lattice fringe (Figure 3D) could be Ru(0){101}/RuO_2_ {210} (see later discussion).

In contrast to the low-Ru sample, regions of elemental overlap were clearly visible in the high-Ru sample (Figure 3E). An enlarged image (Supplementary Figure S5) shows numerous apparent core-shell structures as well as several “twinned” structures of the two metals alongside each other. In addition, a triplet structure (“dumbbell”) is apparent that comprises a pivotal bimetallic region abutting onto separate nanostructures of both Pd and Ru (Supplementary Figure S5). These features contrast with the low-Ru preparation that shows no evident hybrid structures (Supplementary Figure S4).

The electron microscopy data would indicate that, with excess Ru, the material comprises mostly a random deposition of Pd and Ru/RuO_2_ NPs but with some core-shell structures apparent visually. Examination of an area with a small undefined NP shows a largely random distribution of Pd and Ru with metal levels barely detectable above the background at the edge of the NP transect (Figure 3I). In contrast the patterning of a well-defined NP confirms a core-shell structure (Ru core/Pd shell) as described previously for Pd/Au (Au core/Pd shell: Deplanche et al., 2012). The previous studies on Pd/Au NPs also used Z-imaging, where the image intensity reflects the Z dependence on atomic number (Nellist and Pennycook, 2000); this can be used to localize atoms in NPs where elements of higher atomic number appear brighter. In bio-Pd/Au core-shells the Au-core was evident (Tran et al., 2012; Z Pd = 46; Z Au = 79) and, similarly, the Pt in Pd/Pt alloy (Z Pt = 78: Esparza et al., 2017). However, since Z for Ru = 44 (i.e., very close to Pd) the difference in contrast between the metals would be too small to detect. However, Figure 3H provides evidence for the occurrence of a similar structure in bio-Pd/Ru; the mechanism was assigned previously to re-oxidation of the Pd(0) “seeds” via galvanic reduction of the incoming Au(III) and migration of nascent Pd(II) around the NP, to be re-reduced under H_2_ to form the shell around the Au(0) core (Deplanche et al., 2012). In contrast to bio-Pd/Au, the Pd/Ru core-shell structures occurred only occasionally and the occurrence (and persistence) of Ru(0) in the material is not proved (see above and later). Indeed, formation of Ru(IV) as RuO_2_ is suggested (i.e., oxidation of Ru(III) see later) which requires an electron sink. Petkov et al. (2017) attribute electronic interactions at surface metal interfaces (Au^δ+^; Pd^δ–^) as being responsible for high catalytic activity. It would seem possible that, in this case under H_2_ a core-shell may form, followed by (in air) oxidation of the Ru(0) component. It is known that a negatively charged Pd(0) can be formed by accepting electrons (i.e., behaving as a capacitor). Indeed, the capacitance (ability to store charge) of bio-Pd on *E. coli* was measured at 0.5–0.6 microamps in an electrochemical test system (at 20 wt% Pd: Courtney et al., 2016).

However, RuO_2_ can evolve in air from Ru(III) (see above) without addition of a specific oxidant. No precaution was taken to exclude air following harvest of the NPs. It would seem that while Ru(III) may be reduced to Ru(0) into an occasional bimetallic core-shell (as for Pd/Au) it can also become oxidized to Ru(IV) and form RuO_2_ in air. While the core-shell may be stabilized by its Pd-overlay, the side by side NPs would leave Ru with an available surface for evolution into RuO_2_ while having Pd(0) nearby as a possible electron acceptor, a possible benefit of co-localization (Supplementary Figures S4, S5) without actual integration of the two metals.

### Analysis of Bulk Material Using EXAFS: XANES Analysis

X-ray absorption near edge structure (XANES) is an element-specific and local bonding-sensitive spectroscopic technique applied in this study to determine the oxidation state of Ru and Pd in the experimental samples. The analysis is based on relating small shifts (a few eV) in XANES absorption edge energies with the average oxidation state of the central element. Spectra of Pd-foil and Ru-foil are shown in Supplementary Figure S7.

**FIGURE 4:**
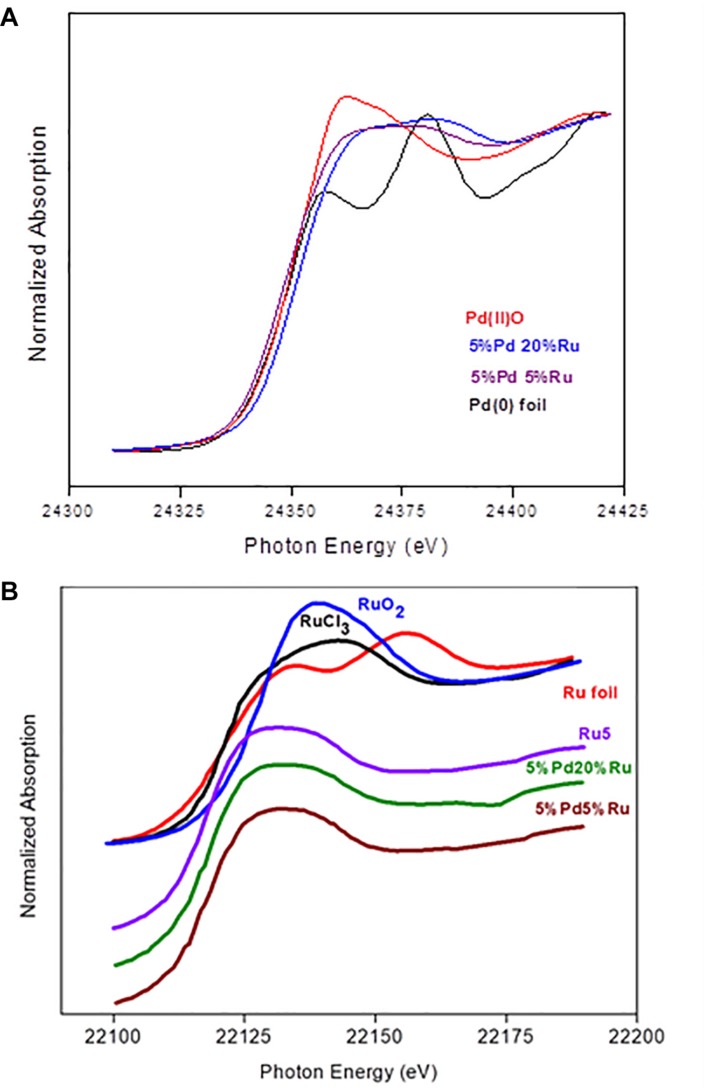
**(A)** XANES region of EXAFS spectra of the Pd K-edge in reference compounds (Pd foil and PdO) and for Pd-Ru biogenic NPs samples. **(B)** XANES spectra of ruthenium foil, RuO_2_, RuCl_3_, 5% Ru, and for Pd-Ru biogenic NPs samples.

Figure 4A shows the XANES spectra of Pd reference compounds; palladium foil (metallic Pd) and PdO [Pd(II)], and biogenic Pd/Ru NPs (low-Ru and high-Ru). The results obtained indicate that Pd is present as a mixture of Pd(0) and Pd(II) in the two Pd/Ru samples. Linear combination fitting mode of ATHENA code was used to determine the relative amounts of Pd(0) and Pd(II) present in the bio-derived samples, revealing a mixture of 60% metallic palladium and 40% Pd(II) for both bulk biogenic Pd/Ru nanoparticles samples.

In the case of the Ru edge (Figure 4B), the XANES spectra of both biogenic NPs samples are different from that of Ru foil. In these samples, Ru is present as a mixture of Ru(III) and Ru(IV). However, linear combination fitting mode of ATHENA code showed the presence of low amounts of Ru(0) ranging between 6 and 10%.

### EXAFS: Pd K-Edge

**FIGURE 5:**
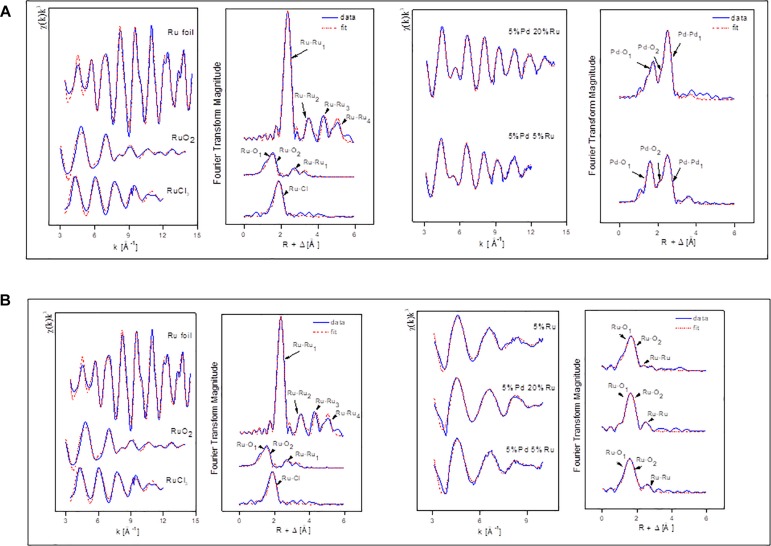
**(A)** EXAFS spectra of Pd foil and Pd-Ru biogenic NPs samples as well as their corresponding FT. **(B)** EXAFS spectra of Ru foil, RuCl_3_, and Pd-Ru biogenic NPs samples as well as their corresponding FT.

The Pd K-edge EXAFS spectra of a palladium foil, and of low-Ru and high-Ru samples, along with their corresponding Fourier transforms (FT), are shown in Figure 5A. The fit parameters of the calculated spectra are summarized in Table 2.

**TABLE 2:** EXAFS structural parameters of the palladium foil and biogenic Pd-Ru NPs.

**Sample**	**Shell**	**N^a^**	**R (Å)^b^**	**σ^2^ (Å^2^)^c^**	**ΔE (eV)**
Pd foil^*^	Pd-Pd_1_	12^d^	2.74	0.0047	–0.66
	Pd-Pd_2_	6^e^	3.86	0.0086	
	Pd-Pd_3_	24^e^	4.78	0.0083	
	Pd-Pd_4_	12^e^	5.40	0.0055	
5% Pd 20% Ru	Pd-O_1_	1.8 ± 0.3	2.10	0.0076	23.2
	Pd-O_2_	1.2 ± 0.1	2.55	0.0076^e^	2.62
	Pd-Pd_1_	3.2 ± 0.4	2.75	0.0084	
	Pd-Pd_2_	1.6^e^	3.85	0.013	
5% Pd 5% Ru	Pd-O_1_	1.5 ± 0.2	2.04	0.0059	14.5
	Pd-O_2_	1.0 ± 0.1	2.55	0.0059^e^	–1.56
	Pd-Pd_1_	3.2 ± 0.3	2.75	0.011	
	Pd-Pd_2_	1.6^e^	3.83	0.014	

^a^Errors in coordination numbers are ±25% and standard deviations as estimated by EXAFSPAK; ^b^errors in distance are ±0.02 Å; ^c^Debye-Waller factor; ^d^fixed for calculation; ^e^Coordination number (N) linked to the N of Pd-Pd_1_ path or to Pd-O_1_.^*^Supplementary Figure S7.

In the case of the Pd foil, the FT peaks of metallic Pd were attributed to four Pd-Pd shells with distances of 2.74, 3.86, 4.78, and 5.40 Å. The major peak corresponds to about twelve Pd atoms at a Pd-Pd interatomic distance of 2.74 ± 0.02 Å as reported by Polizzi et al. (2001).

The EXAFS spectra of both low-Ru and high-Ru biogenic NPs samples are characterized by the presence of two Pd species: one metallic (Pd-Pd) and two complexed (Pd-O) via oxygen atoms to the cell matrix functional groups. For the Pd-O_1_ and PdO_2_ phases, the distances found are comparable to the ones of palladium oxide [Pd(II)O] with a simple tetragonal structure (Borowski, 1997) with Pd-O_1_ contributions at 2.1 ± 0.02 Å and 2.04 ± 0.02 Å for high-Ru and low-Ru samples, respectively, and to Pd-O_2_ bond distance at 2.55 ± 0.02 Å. The distances were calculated using the Pd-O backscattering phase and amplitude functions obtained from PdO crystal structure using the FEFF8 program. The oxygen atoms could have originated from the carboxyl groups of, e.g., aspartic and glutamic acids of the bacterial cells as reported by Fahmy et al., 2006. The interatomic distances obtained for metallic phase contribution were very close to the ones of the metallic foil.

### EXAFS: Ru K-Edge

Figure 5B shows the Ru K-edge EXAFS spectra of a ruthenium foil, RuO_2_, RuCl_3_, low-Ru, high-Ru, and Ru-only samples along with their corresponding Fourier transforms (FT). The structural parameters of the calculated spectra are summarized in Table 3.

**TABLE 3:** EXAFS structural parameters of the ruthenium foil, RuO_2_, RuCl_3_ and biogenic Ru and Pd-Ru NPs samples

**Sample**	**Shell**	**N^a^**	**R (Å)^b^**	**σ^2^ (Å^2^)^c^**	**ΔE (eV)**
Ru foil^*^	Ru-Ru_1_	12^d^	2.67	0.004	–1.81
	Ru-Ru_2_	6^d^	3.78	0.0028	
	Ru-Ru_3_	24^d^	4.68	0.0084	
	Ru-Ru_4_	12^d^	5.35	0.0031	
RuCl_3_	Ru-Cl	5.3 ± 0.3	2.35	0.0059	–2.7
RuO_2_	Ru-O_1_	2^d^	1.87	0.002	1.80
	Ru-O_2_	4^d^	1.99	0.002	
	Ru-Ru_1_	2^d^	3.09	0.0068	
	Ru-Ru_2_	8^d^	3.56	0.016	
5% Ru	Ru-O_1_	1.1 ± 0.1	1.96	0.001	7.5
	Ru-O_2_	2.5 ± 0.2	2.1	0.001^d^	
	Ru-Ru	0.5 ± 0.1	2.85	0.01	
5% Pd 20% Ru	Ru-O_1_	1.8 ± 0.4	2.04	0.001	8.6
	Ru-O_2_	1.4 ± 0.3	2.16	0.001^d^	
	Ru-Ru	1.0 ± 0.2	2.77	0.0012	
5% Pd 5% Ru	Ru-O_1_	1.8 ± 0.1	1.98	0.002	7.4
	Ru-O_2_	1.8 ± 0.2	2.15	0.002	
	Ru-Ru	2.0 ± 0.5	2.77	0.019	

^a^Errors in coordination numbers are ±25% and standard deviations as estimated by EXAFSPAK; ^b^errors in distance are ±0.02 Å; ^c^Debye-Waller factor; ^d^Coordination number (N) linked to the N of Ru-O_1_ path. ^*^Supplementary Figure S7.

The FT of the three experimental samples was well fitted by the use of two Ru-O bonds with interatomic distances of 1.96–2.04 and 2.1–2.16 ± 0.02 Å, and a single Ru-Ru shell with a bond distance of 2.77–2.85 ± 0.02 Å. The distances of the shortest Ru-O bond (1.96–2.04 ± 0.02 Å) can be assigned to Ru=O of RuO_2_ (McKeown et al., 1999) while the shell at bond distance of 2.1–2.16 Å is assigned to the Ru–O_hydroxo_ (Ru–OH) bond as observed in RuNi(OH)_2_ composite (Venkatesan et al., 2009). The EXAFS spectra include also a Ru-Ru shell with a bond distance of about 2.77–2.85 ± 0.02 Å. The higher Debye-Waller factor of this shell (0.01–0.019 Å^2^) indicates that there is probably a wide spread of Ru-Ru distances with an averaged value of 2.77–2.85 ± 0.02 Å. This implies the possible contribution of Ru-Ru arising from two different ligands (Ru metal and RuO_2_). This assumption is supported by two features: (1) the bond distance value of 2.77–2.85 ± 0.02 Å could correspond to the average distance of Ru-Ru from Ru metal (2.66 ± 0.02 Å) and from RuO_2_ (3.09 ± 0.02 Å) obtained for reference compounds (Table 3). These two shells were not represented as separate shells in the FT spectra since their distances span an *R* range that was not large enough to be differentiated as individual peaks in an EXAFS spectrum for which *Δk* = 7 Å^–1^ in agreement with Δ*R* ≥ π/(2Δ*k*) (Merroun et al., 2005); (2) the linear combination fitting results of the XANES spectra suggested the presence of low amounts of Ru metal in addition to Ru(III) and Ru(IV) species (see above).

However, since catalysis would be largely confined to the surfaces of the cells the bulk signal could mask the contributions of the cell surface components, placing minor surface-located species below the level of detection. Hence, the metal composition of the cell surface was investigated using XPS. This surface method probes only the outermost ∼10 nm of the structure, i.e., the depth of the outer membrane and outermost region of the periplasmic space.

### Examination of Cell Surface Bio-Ru and Bio-Pd/Ru by X-Ray Photoelectron Spectroscopy

**FIGURE 6:**
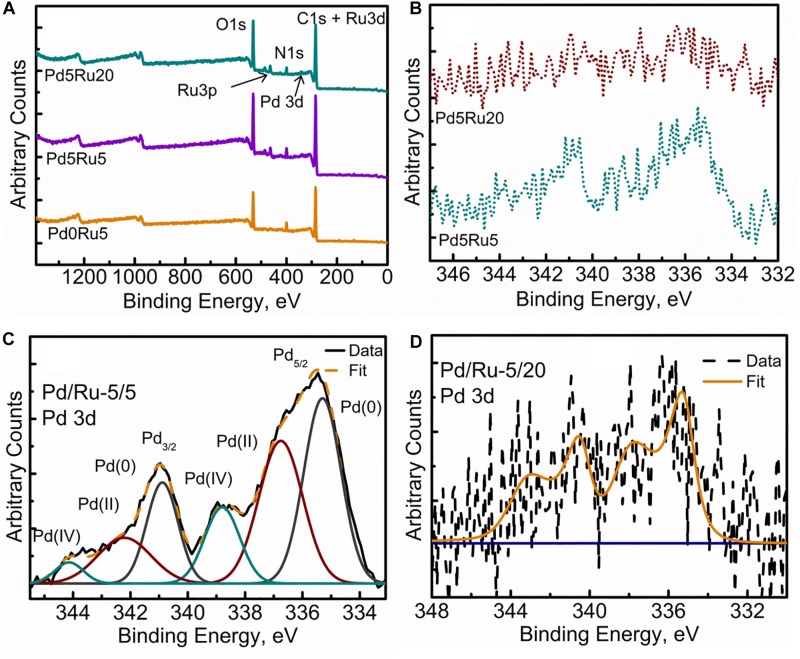
XPS spectra of **(A)** Wide Energy Survey Spectra (WESS) for all three samples, **(B)** high resolution Pd 3d spectra, and fitted components for **(C)** Low Pd and Ru loading sample and **(D)** low Pd high Ru loading sample.

The surface-bound NPs of whole cells (the outermost ∼10 nm of the cell wall) were examined by XPS, where the reduction of Pd(II) to Pd(0) in the “seeding” step was confirmed previously (Omajali, 2015; Omajali et al., 2017). The wide energy spectrum for all samples is shown in Figure 6A. All samples clearly evidenced the presence of the C 1s+Ru 3d peak along with the oxygen O 1s signal centered at ∼285 and ∼530 eV, respectively. Apart from these, the nitrogen N 1s, Ru 3p and, where applicable, low intensity signals of Pd 3d signals were also identified. The spectrum for commercial RuCl_3_ salt (the starting material) is shown in Supplementary Figure S8 for reference, evidencing Ru(III) as RuCl_3_ and Ru(OH)_3_ species.

**FIGURE 7:**
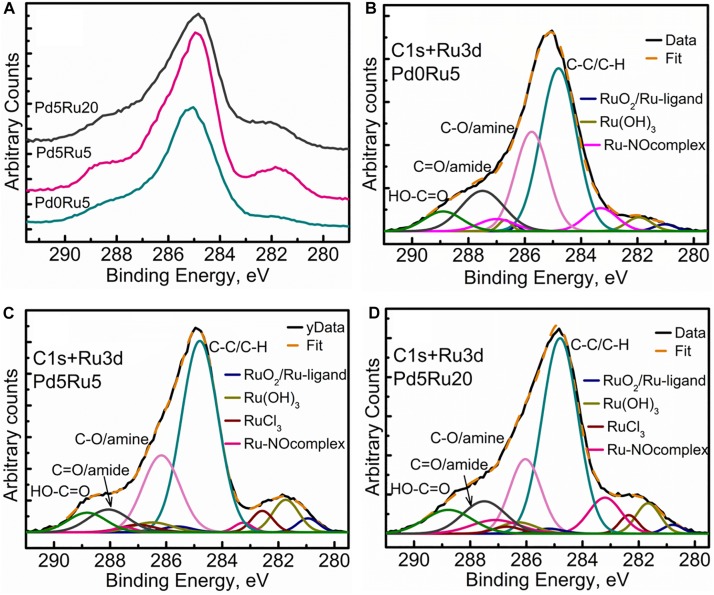
Carbon and Ruthenium XPS spectra showing **(A)** comparison of high resolution C 1s+Ru 3d region for all three samples, **(B)** C 1s+Ru 3d peak fitting for low Ru only sample, **(C)** C 1s+Ru 3d peak fitting for low Pd and low Ru loading sample, **(D)** C 1s+Ru 3d peak fitting for low Pd and high Ru loading sample.

Figure 6B shows a comparison of the high resolution Pd 3d spectra for low-Ru and high-Ru bimetallic samples. Resolved and fitted components for the two samples are shown in Figures 6C,D, respectively. The spectra were fitted using Gaussian peaks to identify the oxidation states of Pd. In low-Ru samples, Pd was found in its native Pd(0) and oxidized Pd(II) and also Pd(IV) states; Table 4 lists the respective binding energies (Liu et al., 2015; Priestley et al., 2015). However, high-Ru samples revealed a very noisy Pd signal (Figure 7D), which has not been resolved into components. However, it can be stated with confidence that at least two components Pd(0) and oxidized Pd(II) are present in these samples. The weak Pd 3d signals could be suggestive of the relatively large amount of Pd internalized by the cells (Figures 2, 3) resulting in minimal Pd nanoparticle formation near the bacterial outer membrane and outermost wall layers which are within the sampling depth of XPS.

**TABLE 4:** XPS peak positions for the various components identified in the high resolution elemental spectra.

	**Binding energies as recorded for different samples, eV**		
	
	**5% Ru**	**5% Pd 5% Ru**	**5% Pd 20% Ru**
**C1 s**			
C-C/C-H	284.8	284.8	284.8
C-OH/amine	285.7	286.0	286.1
C=O/amide	287.5	287.5	288.0
COOH	288.8	288.7	288.8
**Ru3d**			
RuO_2_/Ru-ligand	281.0, 286.3	280.7, 285.2	280.9, 285.6
Ru(OH)_3_	281.9, 286.7	281.7, 286.3	281.7, 286.5
RuCl_3_	–	282.3, 286.7	282.6, 287.0
Ru-NO	283.3, 287	283.3, 287.1	283.2, 287.5
**Pd3d**			
Pd(0)	–	335.3, 340.8	–
Pd(II)	–	336.7, 342.2	–
P(IV)	–	338.7, 344.1	–
**O1s**			
Me-Ox	529.6	529.8	529.8
O=C/sulfates	531.0	531.2	531.3
O-C/O-N	532.2	532.1	532.4
O-C (phenolic)/SiO_2_	533.2	533.2	533.3
H_2_O ads.	534.4	534.4	534.5

Figure 7A shows the comparison of C 1s+Ru 3d high-resolution spectra for all three bio-NP samples. Figures 7B–D show, respectively, low-Ru and high-Ru samples, and Figure 7B the Ru-only sample. The high-resolution C1 s spectra for *E. coli* has been previously resolved into four components identified as C-C (284.5 eV), C-OH/amine (286 eV), C=O/amide (288 eV), and COOH (290 eV), respectively (Priestley et al., 2015). The introduction and growth of metal NPs in the bacterial biomass (Figures 7B–D) revealed a significant change in the spectra, suggesting some loss of C=O/amide and C-OH/amine groups along with the introduction of various Ru components. The amount of Ru contribution to the C 1s+Ru 3d spectra appears to increase as the metal loading increases (from 2.6 wt% Ru only (Table 1) to 5% Ru and 5% Pd to 5% Pd and 20% Ru loading, as seen in Figures 7B–D). This could suggest an interactive behavior enabling a greater amount of Ru to stay near the outer bacterial membrane (or limited/reduced uptake of Ru with increasing Ru loading), in the presence of Pd in the system. Specifically, four C1 s components, namely, C-C/C-OH, C-O/amine, C=O/amide, and COOH were identified/resolved in all three types of metal loaded *E. coli* samples. Three to four types of Ru doublet components were also resolved in all three metal NPs – bacterial biomass samples (Figures 7B–D). These were attributed to RuO_2_, (Morgan, 2015) RuCl_3_, and Ru(OH)_3_. Further analysis of Ru 3p spectra (Supplementary Figure S9) suggested the possibilities of Ru-ligand complexes forming with aromatic carbon structures and Ru-nitrogen oxide (Ru-NOx) like structures being formed within the biological system. Ru complexes are known to be formed within cellular structures, as discussed earlier. These components were, therefore, identified in the Ru 3d spectra, which led to dual attributions, given the minor shifts in the binding energies (Table 4) in comparison to the literature (Morgan, 2015). The presence of Ru-NOx components corroborated with the N 1s spectra (not shown). It is worth mentioning that the RuCl_3_ component could not be distinctly identified in the Ru-only sample. Since this sample has only small quantity of metal added (2.6% Ru only: Table 1) it is possible to have minimal or no RuCl_3_ residues and maximum uptake and internalization of Ru into cellular layers beneath the XPS-accessible depth. The slight shift of Ru(OH)_3_ toward higher binding energy may be due to small residues of RuCl_3_ which could not be resolved into a distinct peak given the complexity of the C 1s+Ru 3d spectra.

**FIGURE 8:**
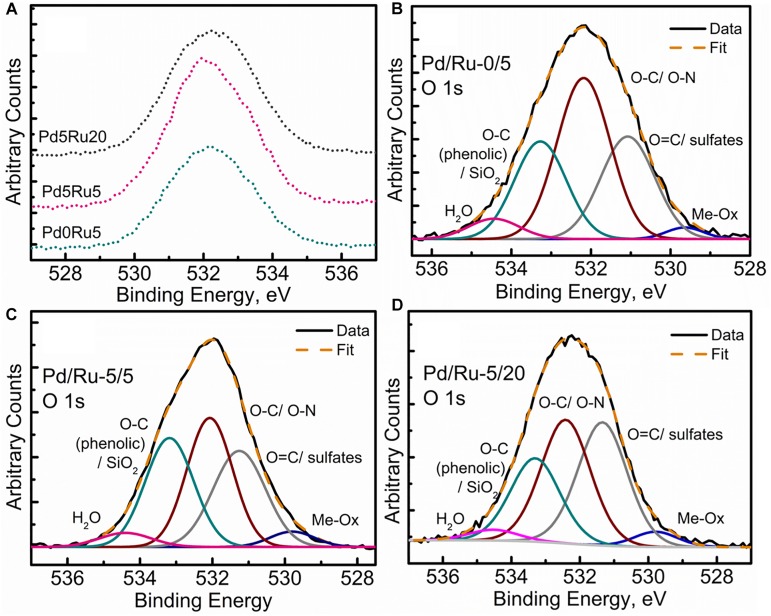
Oxygen XPS spectra showing **(A)** comparison of high resolution O 1s region for all three samples, **(B)** O 1s peak fitting for low Ru only sample, **(C)** O 1s peak fitting for low Pd and low Ru loading sample, **(D)** O 1s peak fitting for low Pd and high Ru loading sample.

The high-resolution O 1s spectra for all samples can be seen in Figure 8A. Specifically, the deconvolution of O 1s spectrum into the various components for all three samples (Figures 8B–D) revealed a small component peak at ∼529.5 eV, suggesting the presence of metal oxides, which is in agreement with the Pd 3d and Ru 3d spectra, with the EXAFS analysis (PdO) and with RuO_2_ evolving from Ru(OH)_3_ (see earlier). Another observation was the decrease in the C-O component and a relative increase in C=O/sulfate components as the metallic content increased in the form of Ru-alone and low-Ru and high-Ru bimetallics. This could be due to singly bonded oxygen being sacrificed for metal (Ru/Pd) oxide formation. A similar trend was also observed for the adsorbed water content peak denoted by the component peak near 534.7 eV. The slight loss of adsorbed water content and its possible implication is not very clear. Perhaps it can be suggested that with an increase in the metallic component, the formation of various Ru oxides and complexes was promoted and contributed to by the adsorbed/loosely bound water molecules within the cellular structures. However, the exact nature of such interactions in such a complex system would be difficult to predict within the limitations of this study.

None of the spectra provide sufficient evidence for the occurrence of Ru(0) as no peaks are visible at 280 eV.

### Catalytic Activity of the Metallized Cells in the Conversion of 5-HMF to 2,5-DMF

Most published work has used commercially available substrates but, since one of the goals of this work is to realize resources from wastes, 5-HMF was extracted from hydrolyzates of starch and cellulose made by thermochemical hydrolysis methods, previously developed to yield parallel fermentable and added-value side-streams (see Introduction). The catalysts were therefore tested against commercial 5-HMF and 5-HMF from hydrolyzate extracts in a common solvent (MTHF) for extraction and catalytic upgrading. Preliminary work established that this solvent extracted between 60 and 65% of the 5-HMF (Orozco, unpublished) but the extraction method was not optimized.

**FIGURE 9:**
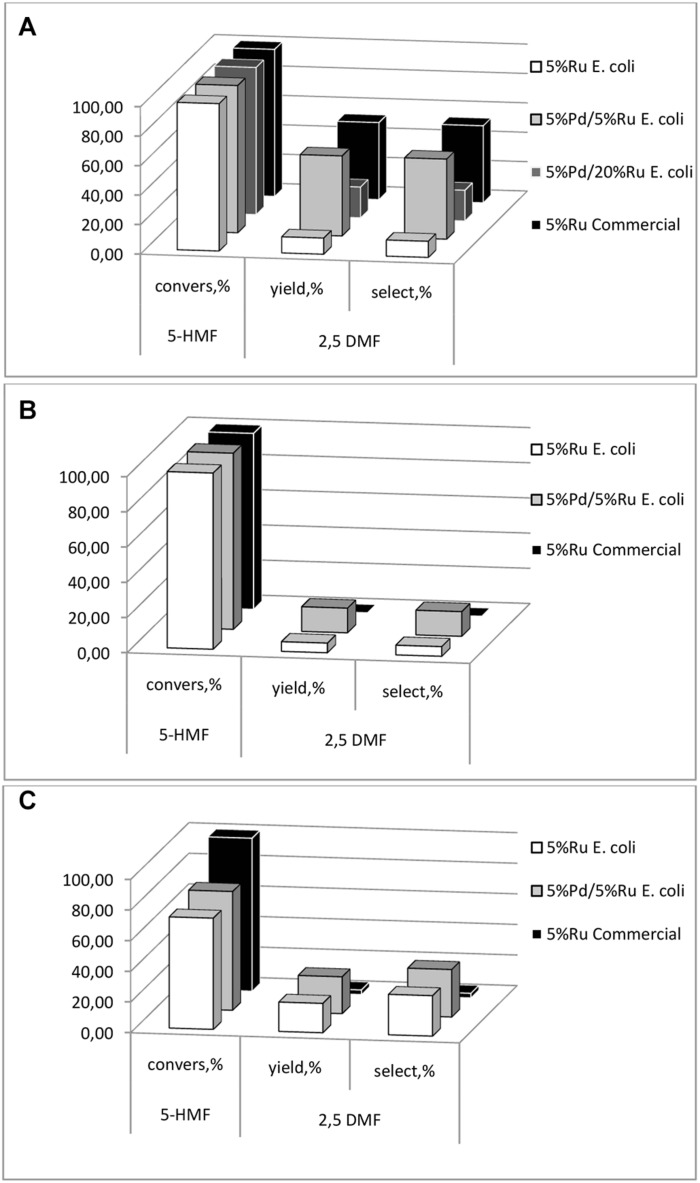
5-HMF conversion, DMF yield and selectivity to DMF of the catalyst preparations using **(A)** synthetic 5-HMF (commercial source), **(B)** starch and **(C)** cellulose hydrolyzates with 5-HMF extracted into MTHF which was also the solvent for the reaction.

First, commercial 5 wt% Ru/C and 5 wt% Pd/C catalysts were compared in MTHF using commercial 5-HMF. The Ru catalyst gave 100% conversion of 5-HMF with 57.1% selectivity to 2,5-DMF with respective values of 100 and 3.3% for the Pd/C equivalent. Therefore, Pd-only catalysts were not considered further. All catalysts tested (data are shown in Supplementary Table 1) gave close to 100% conversion of 5-HMF (Figure 9A) but the high-Ru biomaterial showed a low yield (21%) and selectivity to 2,5-DMF and this, too, was not considered further. It would seem that the presence of the core-shells in the high-Ru sample confers no benefit but these were few in number as compared to the remainder of the metal NPs (Supplementary Figure S5). Lei et al. (2014) noted an adverse effect of an excess of Ru, attributing this to excessive Ru accelerating the occurrence of side reactions. Hence further tests compared the low-Ru samples and 5 wt% bio-Ru.

Using pure 5-HMF the bio-Ru was less effective than the commercial 5 wt% Ru/C catalyst (∼10% selectivity) whereas the low-Ru bimetallic and commercial catalyst gave similar results (>50% selectivity: Figure 9A). However, notably the commercial Ru/C catalyst had little activity against 5-HMF made from the starch or cellulose (Figures 9B,C). It is possible that residual polymeric components fouled the commercial catalyst (co-extracted into the MTHF), or that catalyst poisons were made via the thermochemical hydrolysis reactions, but this was not tested and assumes that these adverse components were co-extracted into MTHF. An alternative explanation is that the chemical catalyst was over active in this reaction, proceeding into additional by-products. An example of the product mix is shown in Supplementary Information (Supplementary Figure S10). In each case (Figures 9B,C) the 5 wt% Pd/5 wt% Ru bio-catalyst achieved significant production of 2,5-DMF and, in the case of cellulose hydrolysate, the Ru-only bio-catalyst became comparable to the low-Ru bimetallic (c.f. Figure 9A). A detailed investigation of the reaction pathway was beyond this scoping study and is in progress but clearly the bio-derived catalyst is able to compete effectively with commercial catalyst against pure 5-HMF and outperforms the latter in conversion of 5-HMF from actual hydrolyzates.

## Conclusion

This study shows clearly that bio-Pd/Ru catalyst (5 wt% of each metal) has potential in the production of 2,5-DMF from 5-HMF from biomass hydrolyzate, which is not achieved using a commercial counterpart. This study reports the formation of Pd/Ru core shell structures in an analogous way to those of Pd/Au reported previously but these may contribute little overall to the outcome since the dominance of the non-core-shell excess Ru in the high-Ru material is counterproductive to 2,5-DMF selectivity. It is far from clear what metal species (or combination of them) actually catalyzes the reaction; evidence was found for various valences of both metals but not, equivocally for Ru(0) whereas Ru(IV) was evident (as RuO_2_) [along with Pd(O) and Pd(0) and also Pd(IV)]. The metal speciation is important, e.g., the degree of Ru oxidation was found to influence the catalytic activity in bimetallic Pt/Ru nanoparticles (Wang et al., 2016). It is likely that heterogeneous NPs are produced since the bacterial cell surface offers a variety of ligands to initiate the nucleation of Pd(II) (and also sites that would complex with incoming Ru(III)) and NP evolution may also be influenced by the surrounding biochemical matrix as well as oxygen ingress during catalyst storage in air. Future work would use systems biology approaches to “dissect” the microbial features that contribute to, and steer, metallic NP development. Initial steps have been taken in the case of monometallic Pd-NPs (Torgeman, 2017) that would underpin understanding of bimetallic systems following Pd(0) nucleation.

## Author Contributions

JG-B and IM made and characterized the biomaterials. RO developed the method for 5-HMF extraction from hyrolyzates. RO and JG-B did catalytic testing, with analysis of products by RH. JG-B and MM performed the high resolution SEM/TEM/elemental mapping. MW acquired the XPS. MW and SS performed the XPS interpretations. DB performed the EXAFS measurements with interpretation by MM. The manuscript was authored by LM with all authors contributing to manuscript preparation.

## Conflict of Interest Statement

The authors declare that the research was conducted in the absence of any commercial or financial relationships that could be construed as a potential conflict of interest.
