# Supplementary material for: Synthesis of Pd/Ru Bimetallic Nanoparticles by Escherichia coli and Potential as a Catalyst for Upgrading 5-Hydroxymethyl Furfural Into Liquid Fuel Precursors
Source: Front Microbiol. 2019 Jun 20;10:1276. doi: 10.3389/fmicb.2019.01276 (PMC6595500; doi:10.3389/fmicb.2019.01276)
Supplement: Supplementary file 1 [file Data_Sheet_1.pdf]

Figure S1 Formation of core-shell Pd/Au nanoparticles by *E. coli*

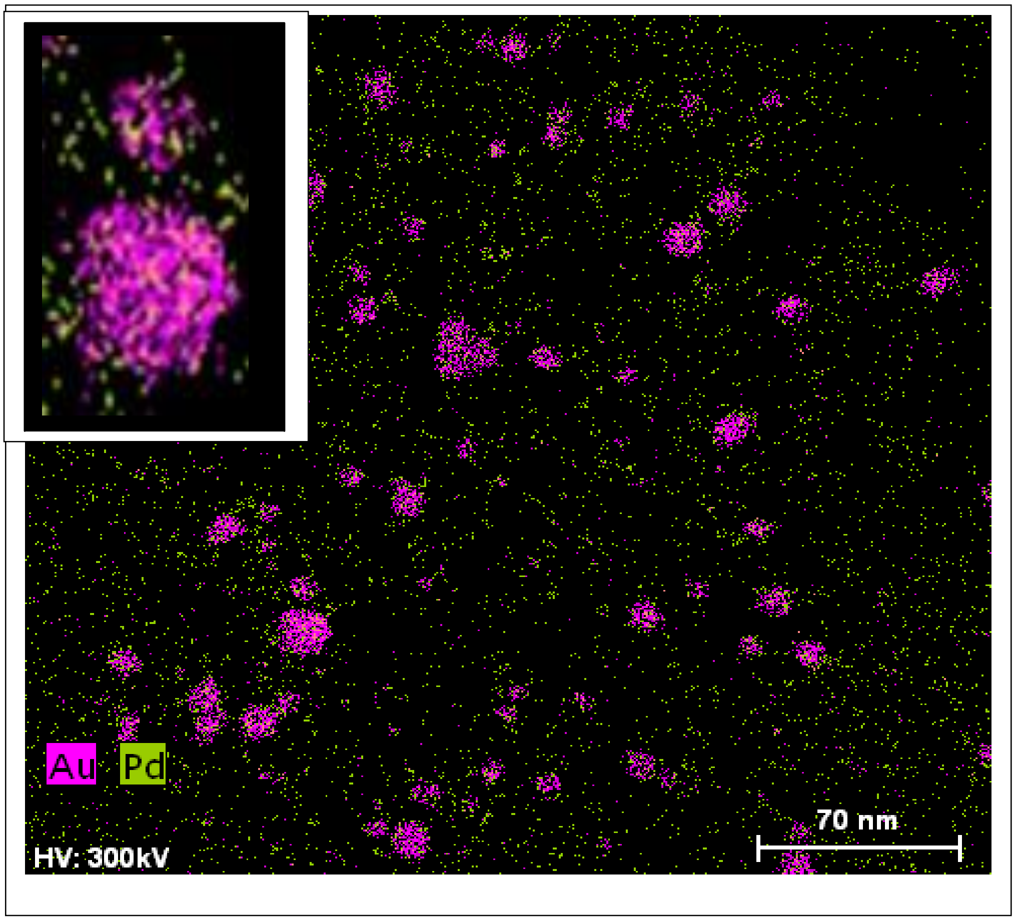

S1: Pd/Au nanoparticles were made according to Deplanche et al (2012). Elemental mapping by EDX (see text) shows atom-arrangements of Pd and Au. Note the Au-core and some intermixing of Au and Pd in the surface layers of the NPs. Loading was 2.5wt%Pd/2.5wt%

Figure S2 Formation of Pd(0) nanoparticles by *E. coli*

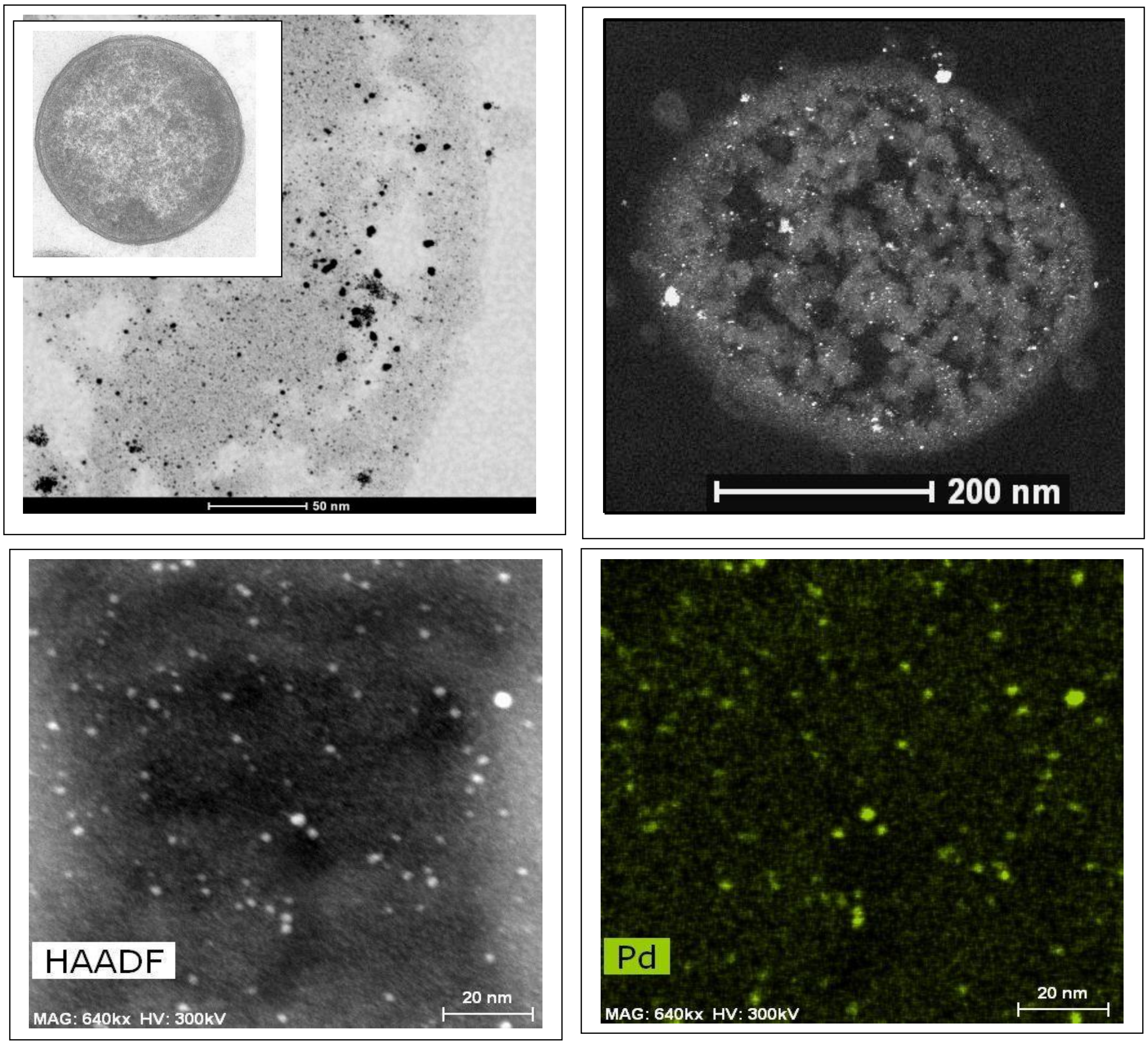

S2: High-resolution HAADF-STEM (High-Angle Annular Dark Field-Scanning Transmission Electron Microscopy) micrographs of *E. coli* (sections) loaded to 5wt%Pd. A: TEM image. B: Dark field image. C:,D: high resolution image with elemental mapping of Pd by EDX.

Figure S3. *E. coli* cells loaded with nominally 20wt% Ru

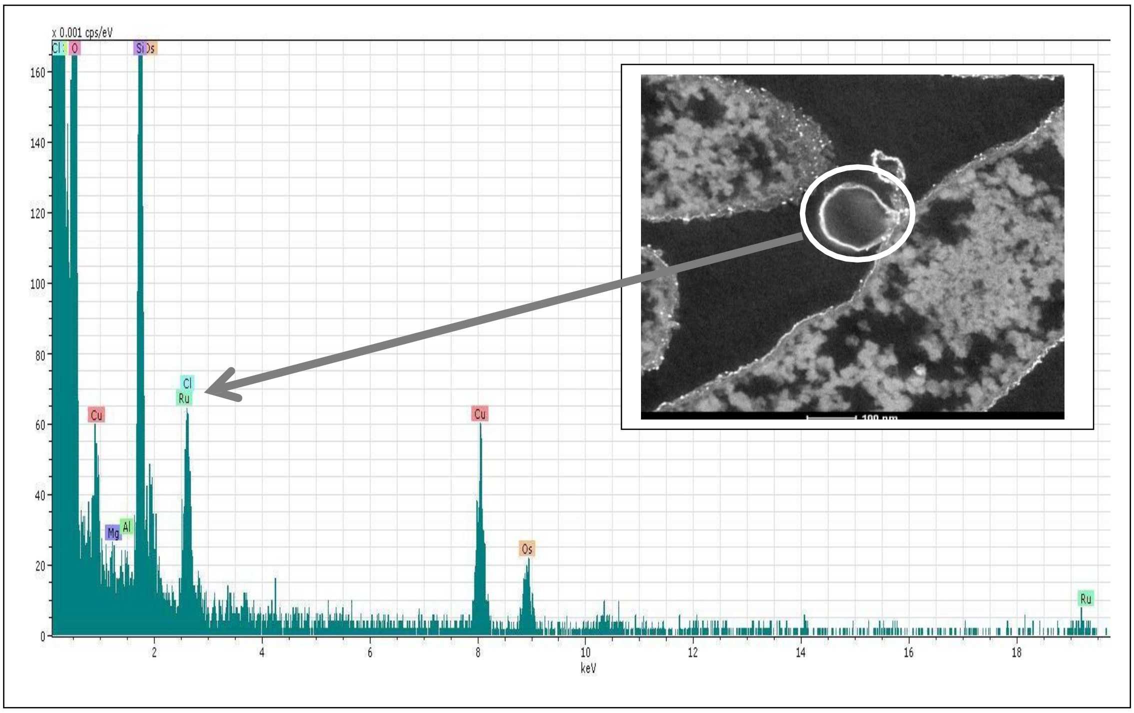

S3: Extruded membrane vesicle from *E. coli* MC4100 loaded with 20wt% Ru (as $RuCl_3$). Note that the X-ray energies of Ru and Cl overlap and hence it is not possible to differentiate easily between sorbed $RuCl_3$ and other species of Ru. However XPS data rule out Cl interference in the Ru peak (see text).

Figure S4. Enlarged image of low-Ru sample

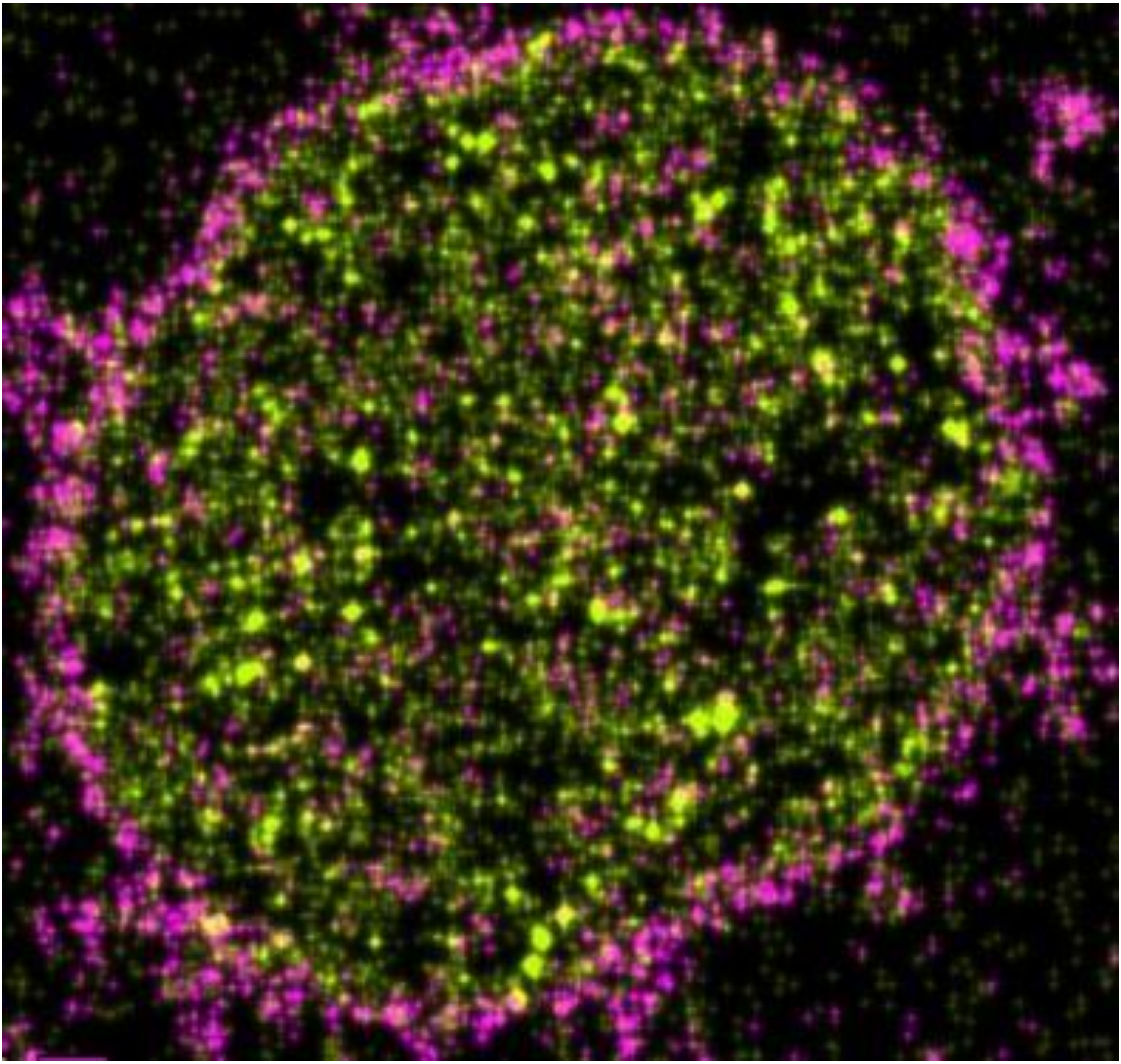

S4: Legend Cross section of single cell loaded to 5wt%Pd/5wt%Ru. Cell diameter is ~ 500 nm.

Figure S5. Enlarged image of high-Ru sample

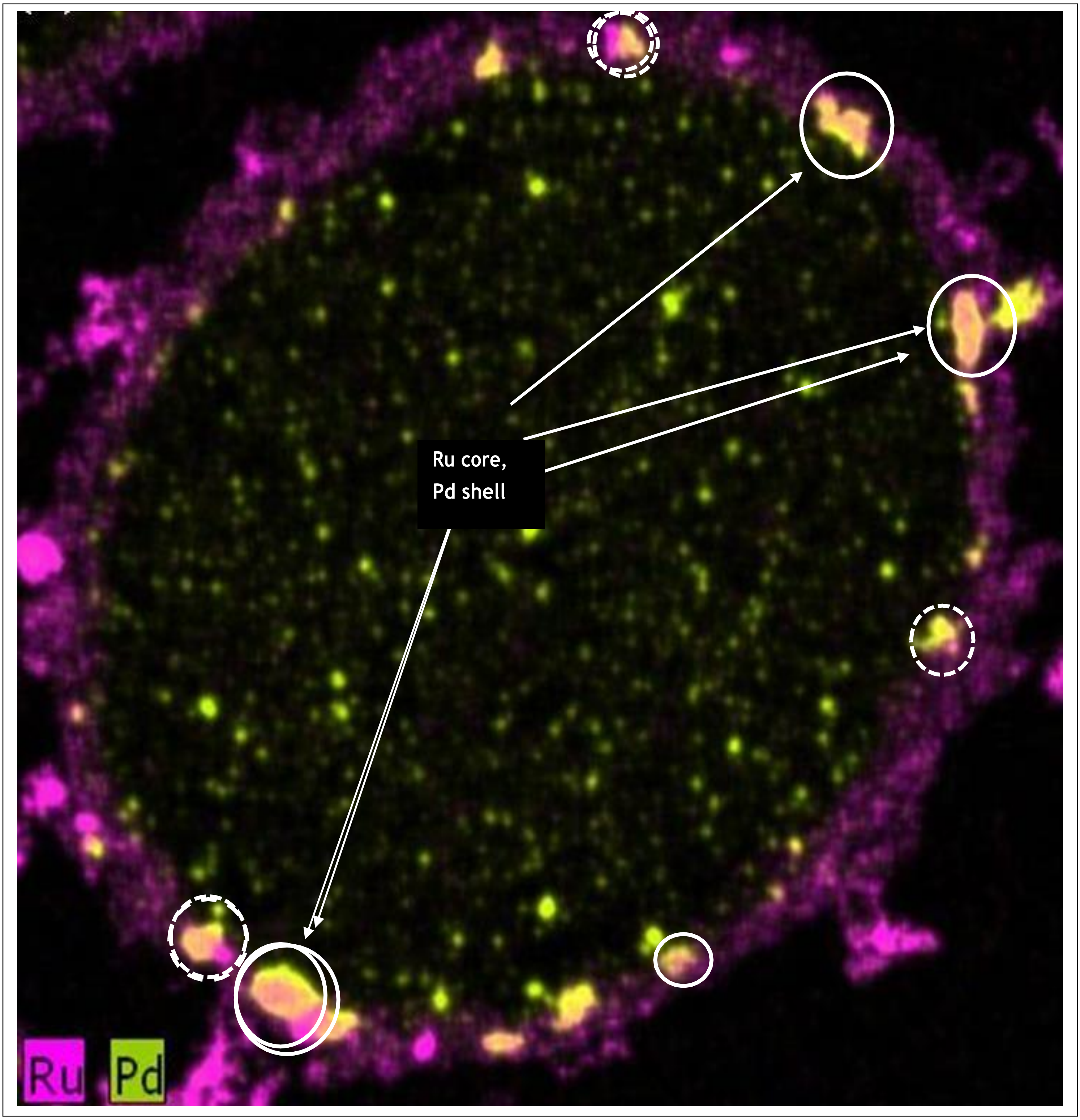

S5: Cross section of single cell loaded to 5wt%Pd/20wt%Ru. Cell diameter is ~ 500 nm. Several core-shell structures are visible (Ru core, Pd shell) (soldi circles) as well as 'twined' structures (dashed circle). A hybrid is also visible that has a triplet structure- a Pd/Ru hybrid area that is twinned with both Pd and Ru (dotted circle).

Figure S6. EDX analysis of small and large intracellular NPs loaded to 5 wt%Pd/20wt%Ru

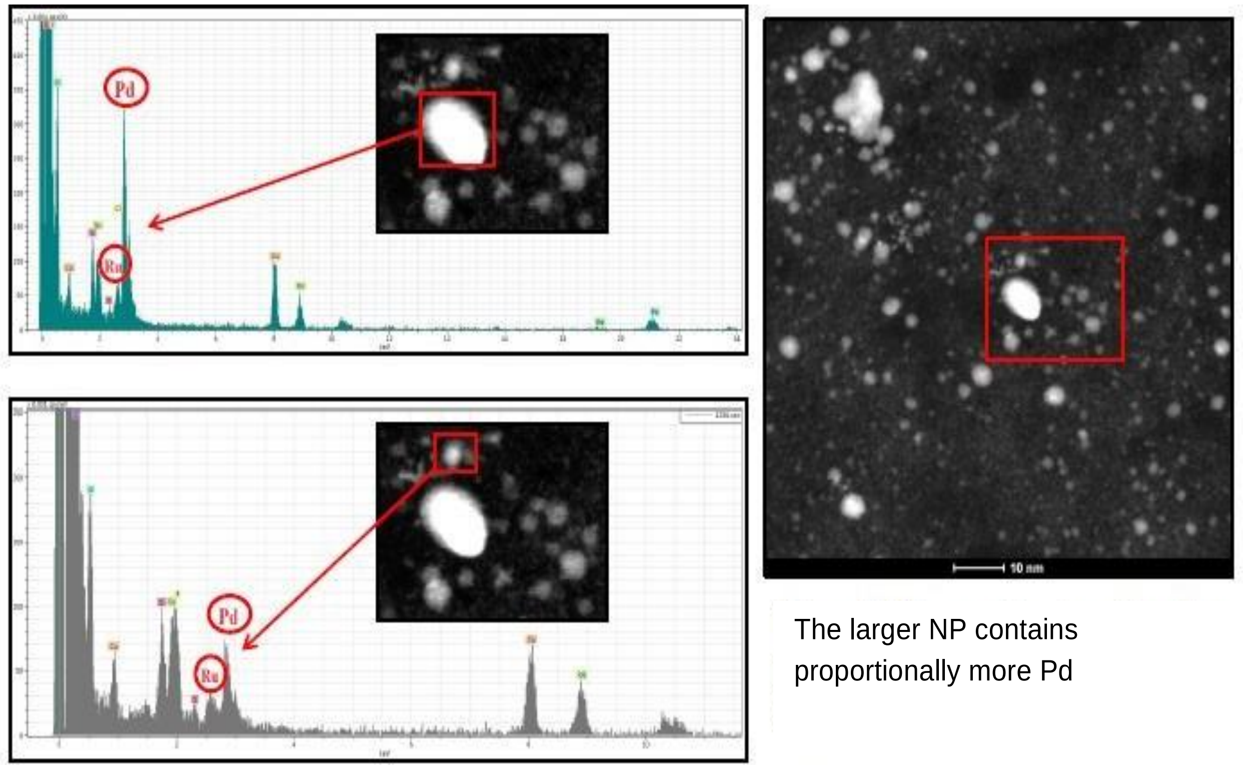

S6: Note presence of Pd and Ru in both large and small NPs.

Fig. S7. EXAFS analysis of Pd-foil

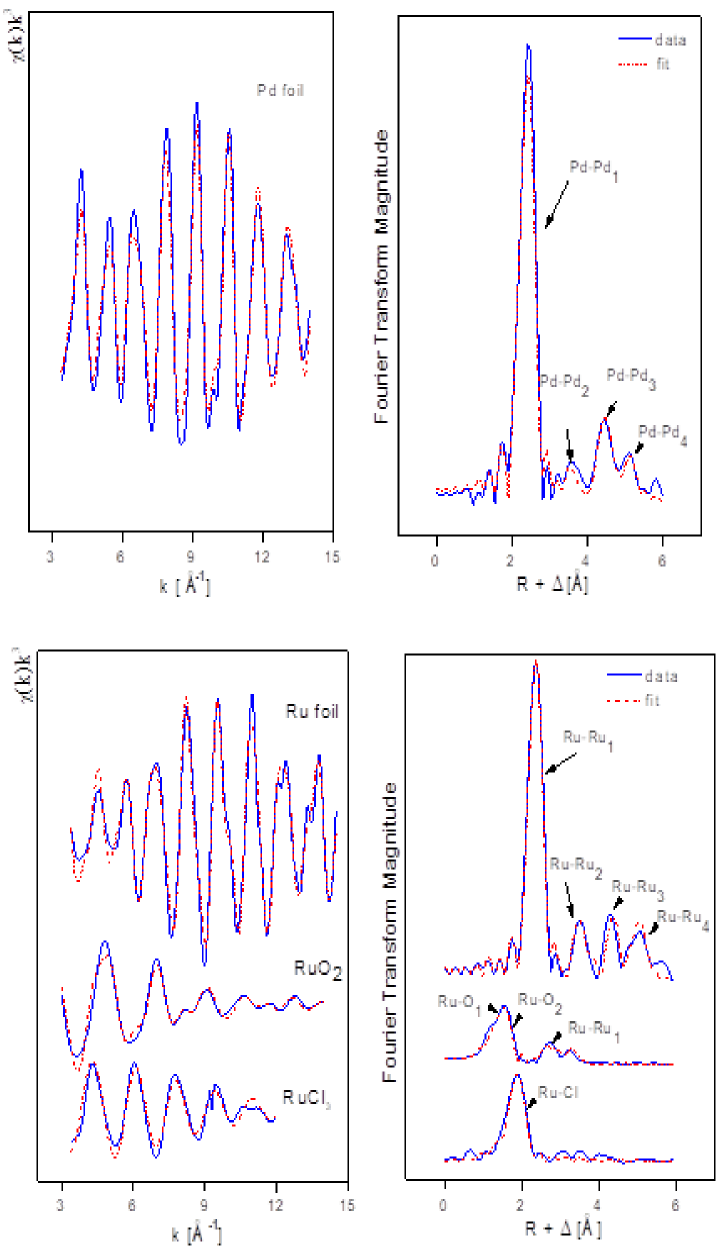

Figure S8: XPS spectra for reference $RuCl_3$ powder used for sample preparation. (Scientific Reports, Omajali et al. in submission)

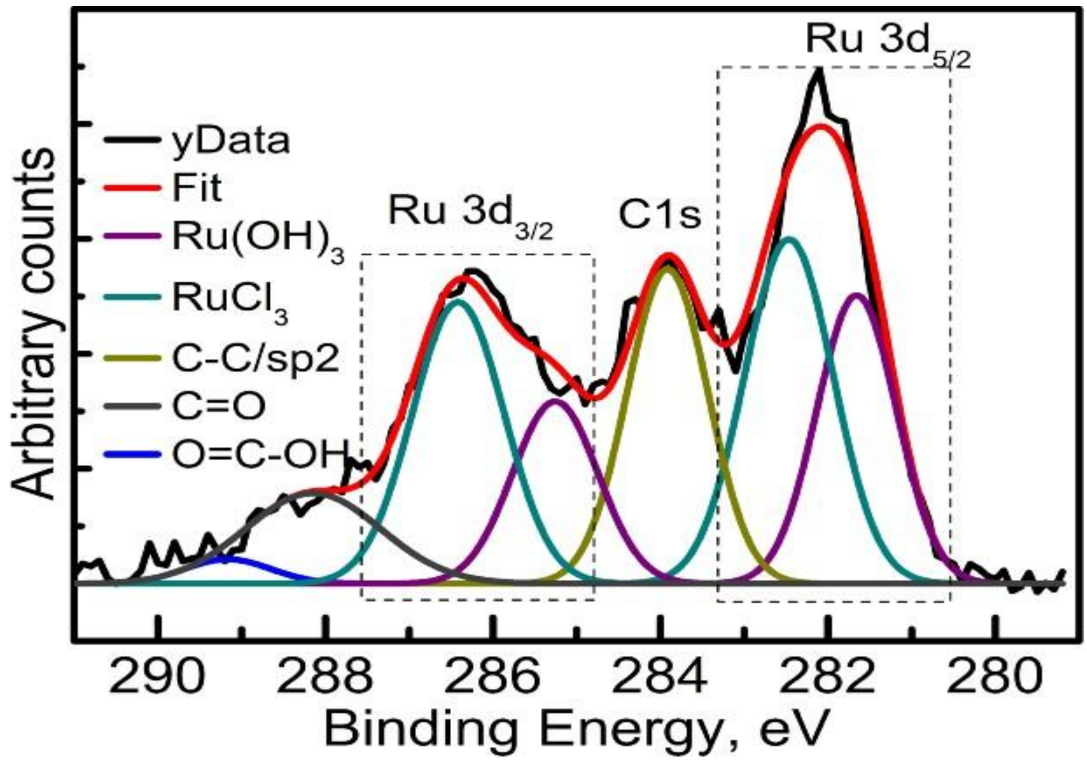

Figure S9: (a) Comparison of the high resolution Ru 3p region for all three samples, (b) Ru $3p_{3/2}$ spectra of low Pd and low Ru loading sample deconvoluted into its various component peaks.

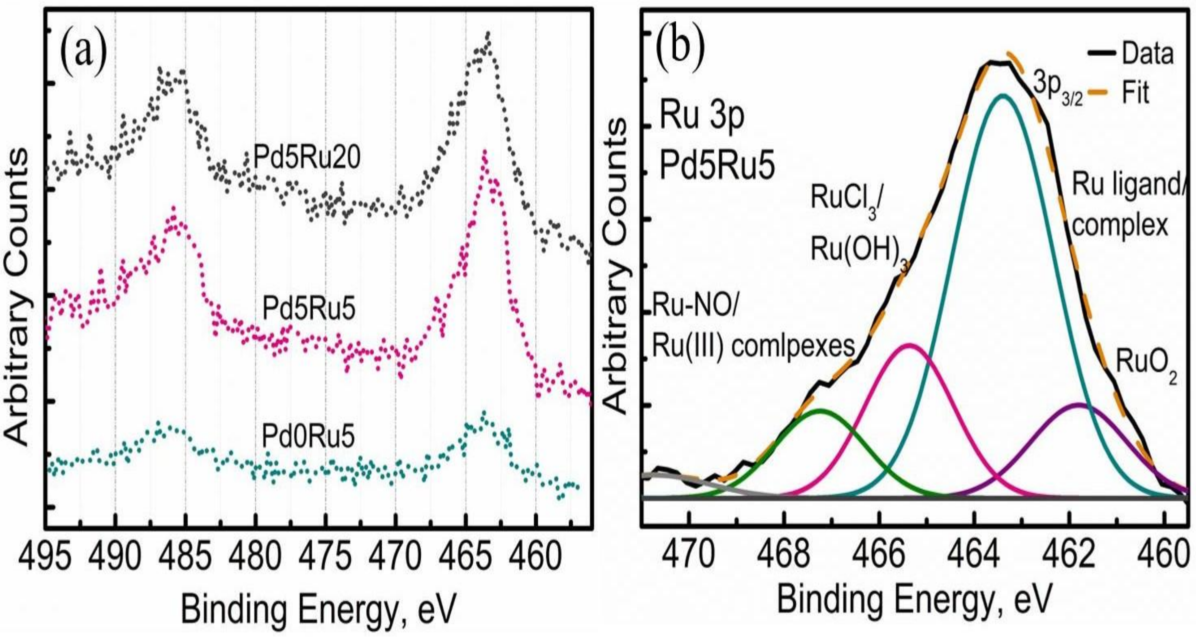

Figure S8a shows the comparison of the high resolution Ru 3p region for all three samples. Figure S8b shows the Ru $3p_{3/2}$ spectra resolved into four components. The deconvolution was carried out on the basis of available literature (Morgan, 2015). $RuCl_3$ and $Ru(OH)_3$ have been attributed to a single peak close to 464.5 eV, following the literature. Similar to those in Ru 3d spectra, other components were also identified in the Ru $3p_{3/2}$ region although some were at slightly higher binding energies than those reported in the literature. Due to lack of literature data on Ru $3p_{1/2}$ component peaks and deconvolution, the deconvolution of the same has not been reported in this study.

Fig. S10. Example chromatogram showing products of catalytic upgrading of 5-HMF extracted from thermochemical hydrolysis of starch

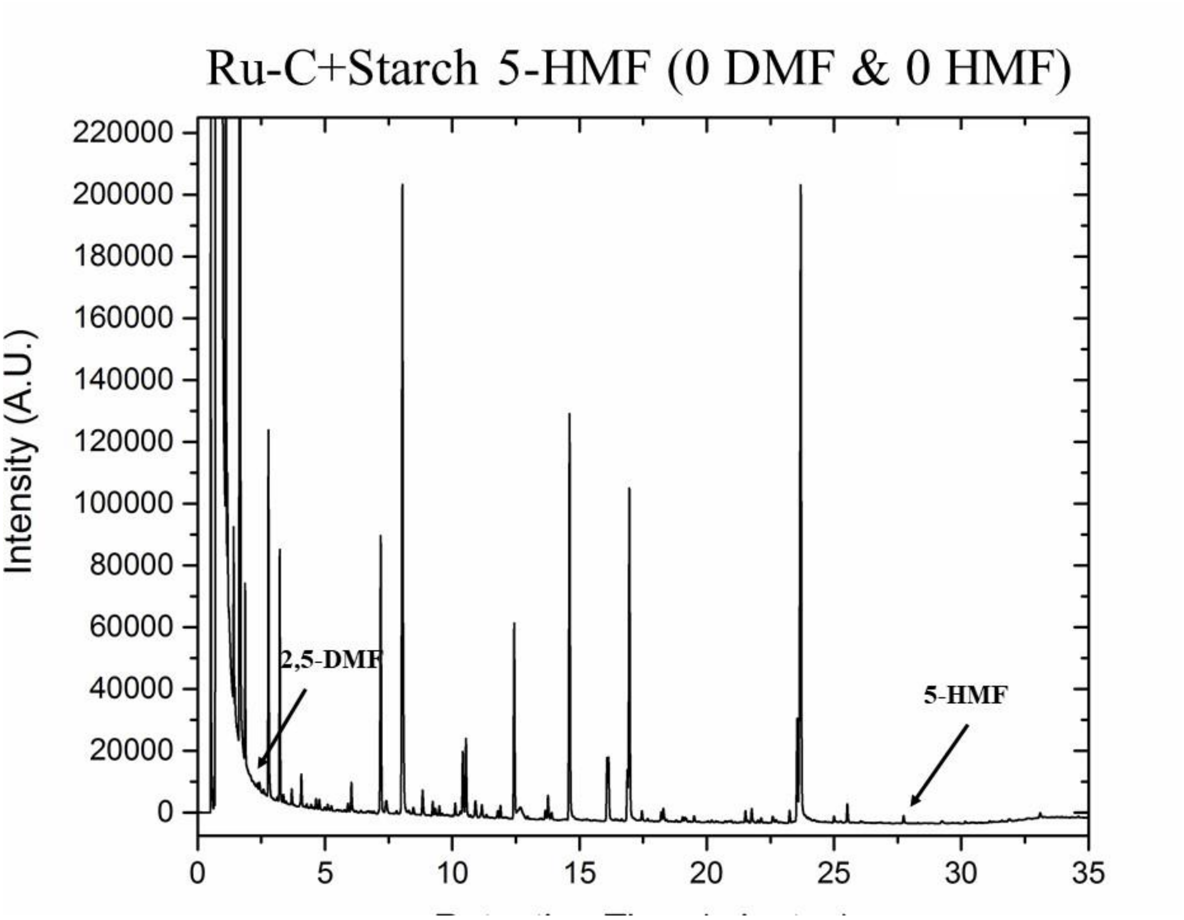

Table 1. Hydrogenation of synthetic 5-HMF, starch and cellulose into 2,5-DMF over different metal loadings of *E.coli* MC4100.

	**5-HMF commercial (Set 1)**			**5-HMF starch (Set 2)**			**5-HMF cellulose (Set 3)**		
**Catalyst**	**5-HMF conversion (%)**	**2,5-DMF yield (%)**	**2,5-DMF Selectivity (%)**	**5-HMF conversion (%)**	**2,5-DMF yield (%)**	**2,5-DMF Selectivity (%)**	**5-HMF conversion (%)**	**2,5-DMF yield (%)**	**2,5-DMF Selectivity (%)**
MC4100 5% Ru	100	10.95	10.95	100.00	5.49	5.49	72.57	19.14	26.38
MC4100 5%Pd/5% Ru	100	54.38	54.38	100.00	14.00	14.00	77.57	24.05	31.00
MC4100 5%Pd/20 %Ru	100	20.9	20.9	NOT DONE					
5% Ru on Carbon	100	52.38	52.38	100.00	0.00	0.00	100.00	3.00	3.00
